# Supplementary material for: Tau follows principal axes of functional and structural brain organization in Alzheimer’s disease
Source: Nat Commun. 2024 Jun 12;15:5031. doi: 10.1038/s41467-024-49300-2 (PMC11169286; doi:10.1038/s41467-024-49300-2)
Supplement: Supplementary file 1 — Supplementary Information [file 41467_2024_49300_MOESM1_ESM.pdf]

Tau follows principal axes of functional and structural brain organization in Alzheimer's disease

Julie Ottoy[1], Min Su Kang[1], Jazlynn Xiu Min Tan[1], Lyndon Boone[1], Reinder Vos de Wael[2], Bo-yong Park[3,4], Gleb Bezgin[5,6], Firoza Z. Lussier[5,7], Tharick A. Pascoal[7], Nesrine Rahmouni[5], Jenna Stevenson[5], Jaime Fernandez Arias[5], Joseph Therriault[5], Seok-Jun Hong[8], Bojana Stefanovic[1,9], JoAnne McLaurin[1,10,11], Jean-Paul Soucy[2], Serge Gauthier[5], Boris C. Bernhardt[2], Sandra E. Black[1,12], Pedro Rosa-Neto[2,5], Maged Goubran[1,9,13]

[1]Sunnybrook Research Institute, University of Toronto, Toronto, ON, Canada

[2]McConnell Brain Imaging Centre, Montreal Neurological Institute and Hospital, McGill University, Montreal, Quebec, Canada

[3]Department of Data Science, Inha University, Incheon, Republic of Korea

[4]Center for Neuroscience Imaging Research, Institute for Basic Science, Suwon, Republic of Korea

[5]Translational Neuroimaging laboratory, McGill Centre for Studies in Aging, McGill University, Montreal, QC, Canada

[6]Neuroinformatics for Personalized Medicine lab, Montreal Neurological Institute, McGill University, Montréal, QC, Canada

[7]Department of Psychiatry, University of Pittsburgh, Pittsburgh, PA, USA.

[8]Department of Biomedical Engineering, Sungkyunkwan University, Suwon, Republic of Korea

[9]Department of Medical Biophysics, University of Toronto, Toronto, ON, Canada

[10]Department of Laboratory Medicine and Pathobiology, University of Toronto, Toronto, ON, Canada

[11]Biological Sciences Platform, Sunnybrook Research Institute, University of Toronto, Toronto, ON, Canada

[12]Department of Medicine (Division of Neurology), University of Toronto, Toronto, ON, Canada

[13]Physical Sciences Platform, Sunnybrook Research Institute, University of Toronto, Toronto, ON, Canada

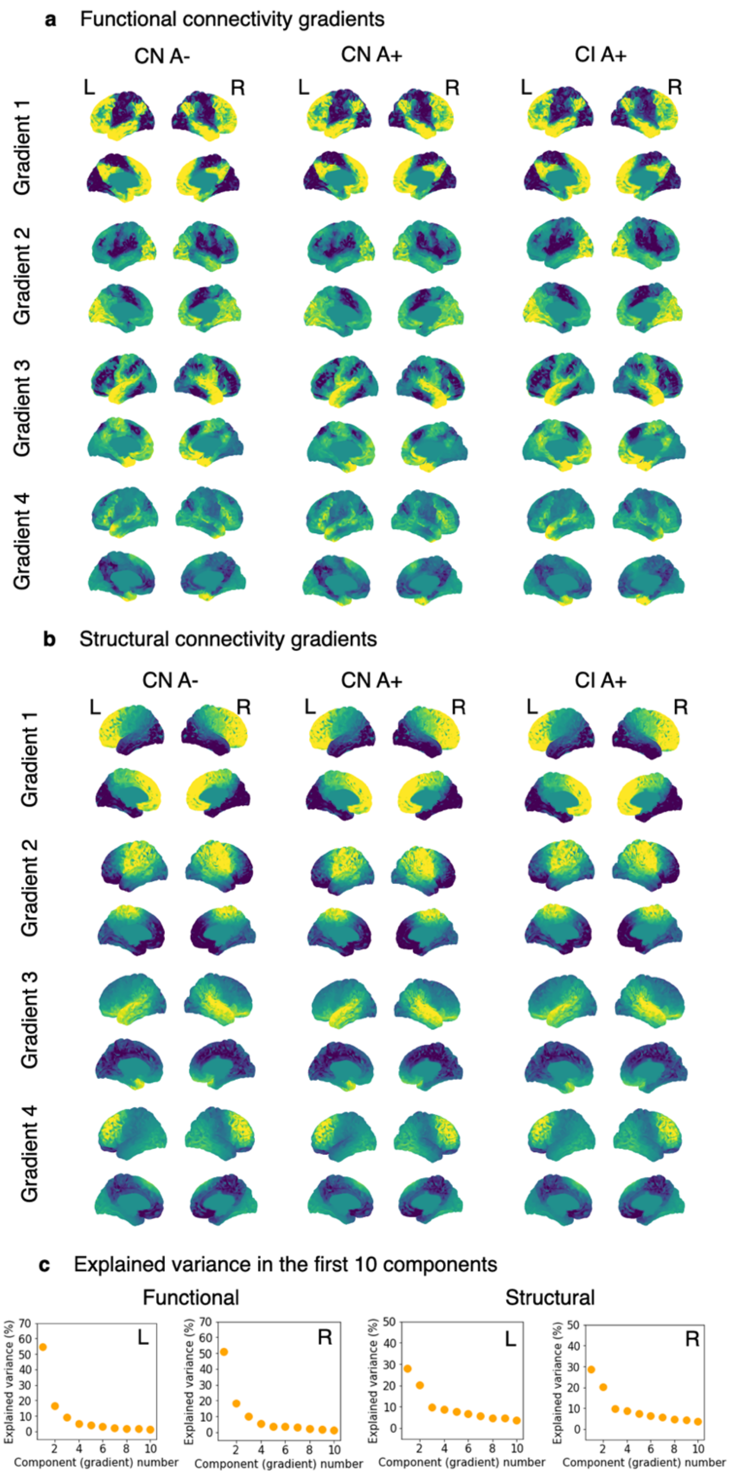

Supplementary Figure 1. **Functional and structural gradient patterns across diagnostic groups**. Panels **a** and **b** show the first four functional and structural connectome gradients, respectively, derived from the group-averaged connectomes (CN A- [n=103], CN A+ [n=35], and CI [n=75]). Panel **c** shows the explained information in the connectome data across the first ten cohort-level gradients for the left and right hemisphere. Source data are provided as a Source Data file.

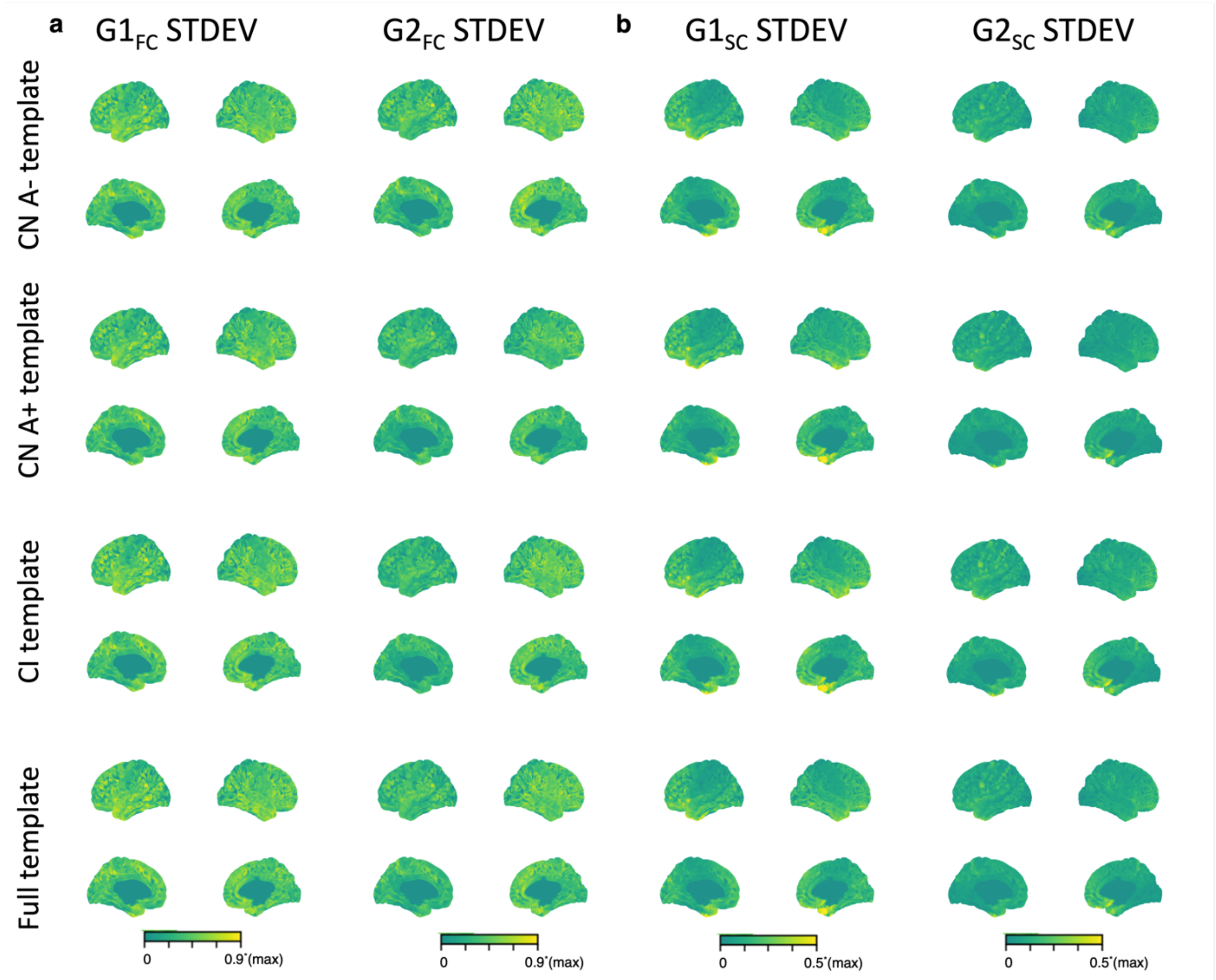

Supplementary Figure 2. Inter-subject variability in the primary functional and structural gradients. Standard deviation (STDEV) maps of subjects' gradient scores relative to their template gradient (per diagnostic group template, see rows 1-3; or per the full cohort template, see row 4). **a,** $G1_{FC}$ showed moderate variability, with the highest and lowest STDEV in the transmodal and unimodal regions, respectively. **b,** $G1_{SC}$ showed low variability, with the highest STDEV in the medial temporal lobe. The resulting maps were stable across template selection and diagnostic groups. The color bar ranges between 0 and 0.9 or 0.5 fraction, respectively, of the maximum STDEV value. Source data are provided as a Source Data file.

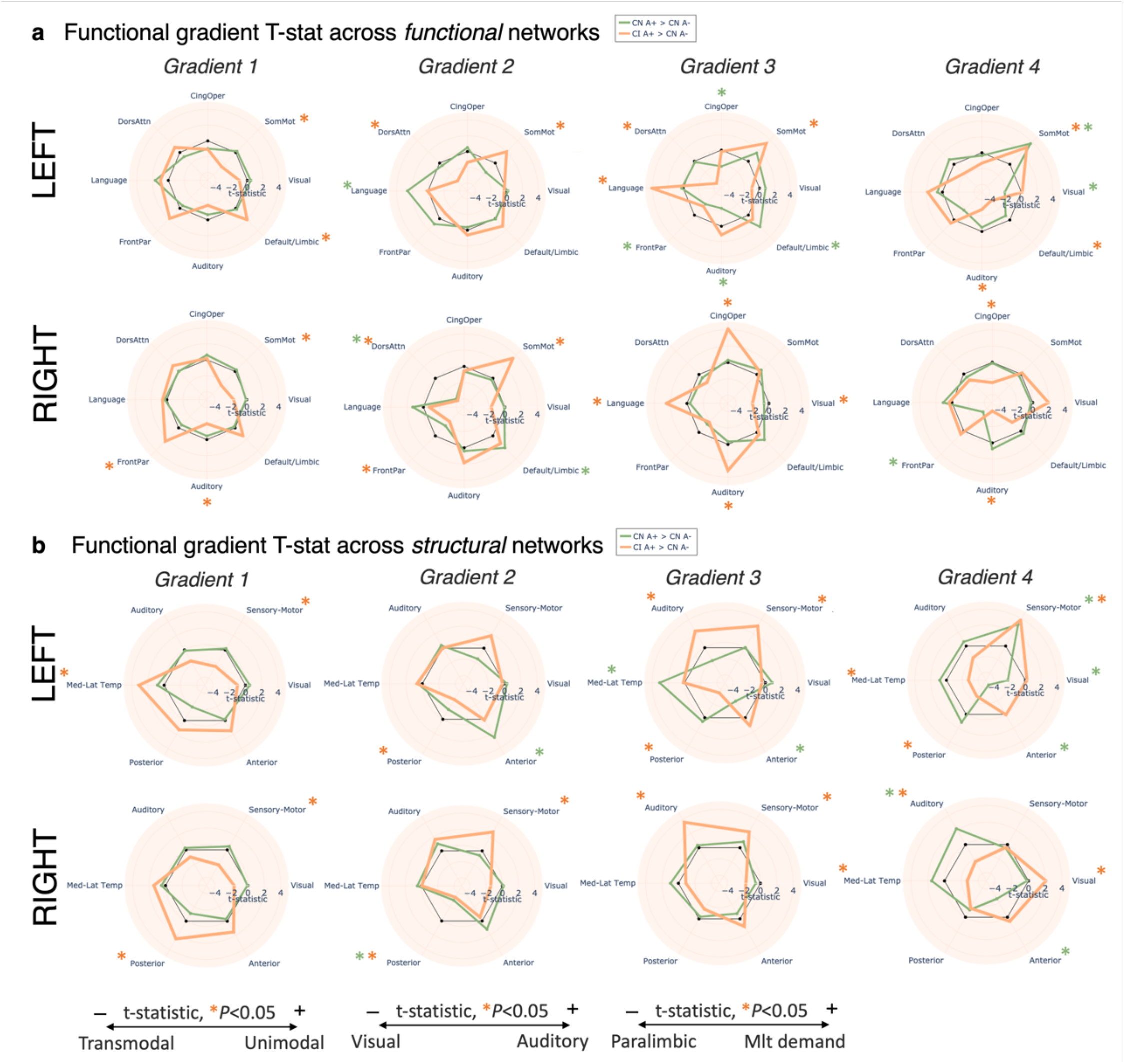

Supplementary Figure 3. Group-wise differences in network-based <u>functional</u> gradient scores aligned to group-wise gradient templates. Spider plots of the group-wise t-statistic differences in network-based gradient scores derived from the individual functional connectomes aligned to group-wise gradient templates. Panels **a** and **b** show the spider plots based on functional (Schaefer) networks and structural (Glasser) networks, respectively. Green and orange lines indicate CN A+ [n=35] > controls [n=101] and CI [n=72] > controls [n=101], respectively. The direction of the positive and negative t-statistic on the gradient is indicated at the bottom of the figure (e.g., regions with a negative t-statistic on $G1_{FC}$ moved closer towards the transmodal cortex in disease compared to controls). Asterisks represent significant t-statistics at the network-level with two-sided $P<0.05$, adjusted for age, sex, and *APOE*-ε4. Source data are provided as a Source Data file.

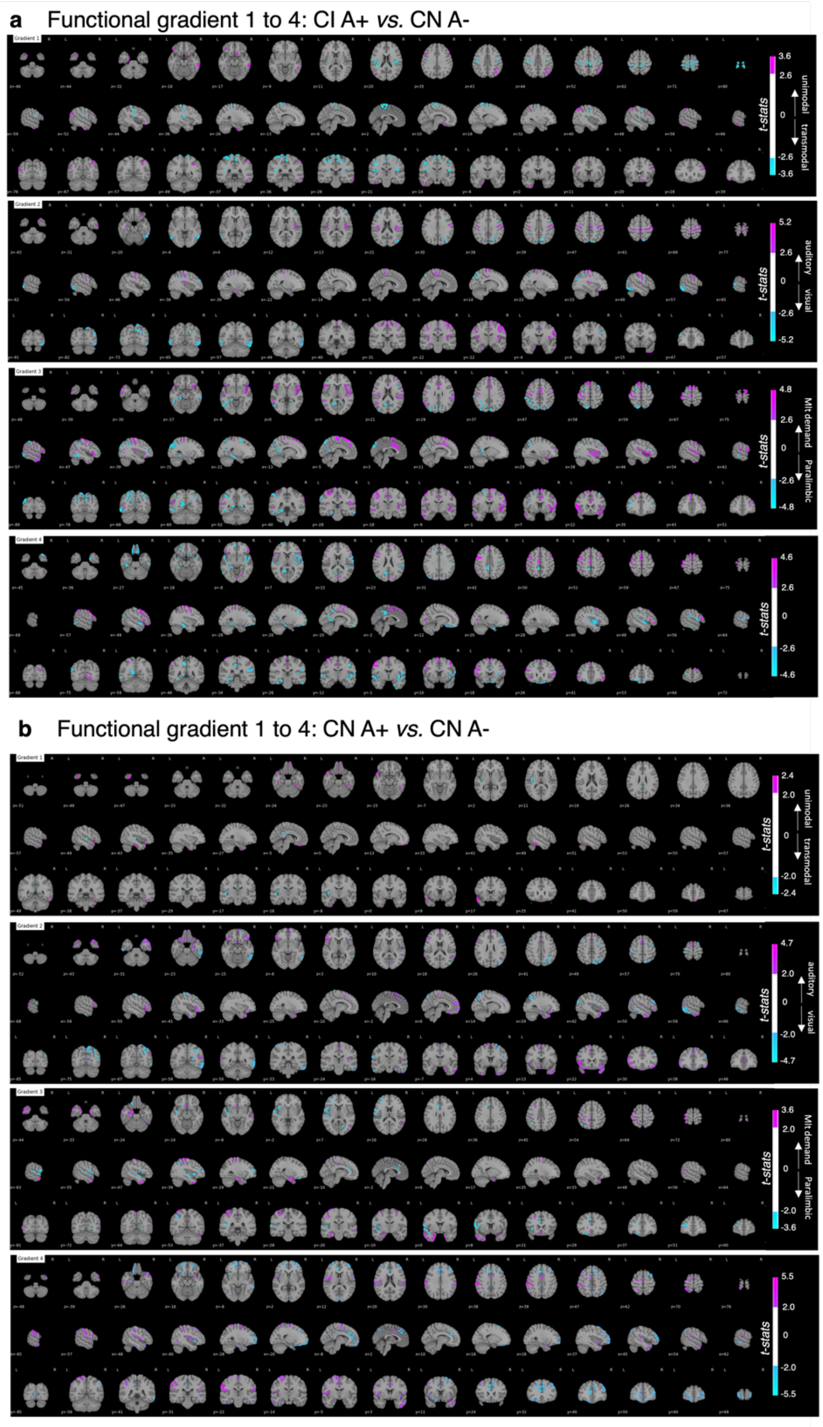

Supplementary Figure 4. Group-wise differences in brain-wide <u>functional</u> gradient scores aligned to group-wise gradient templates. Group-wise t-statistic differences in gradient scores derived from the individual functional connectomes aligned to group-wise gradient templates. Panels **a** and **b** show CI [n=72] > controls [n=101] and CN A+ [n=35] > controls [n=101], respectively. The direction of the positive and negative t-statistic on the gradient is indicated at the right side of the figure. T-statistics were adjusted for age, sex, and *APOE*-ε4, and family-wise errors due to multiple comparisons with a false-positive rate at two-sided $P<0.01$ and cluster-wise threshold of 500 voxels.

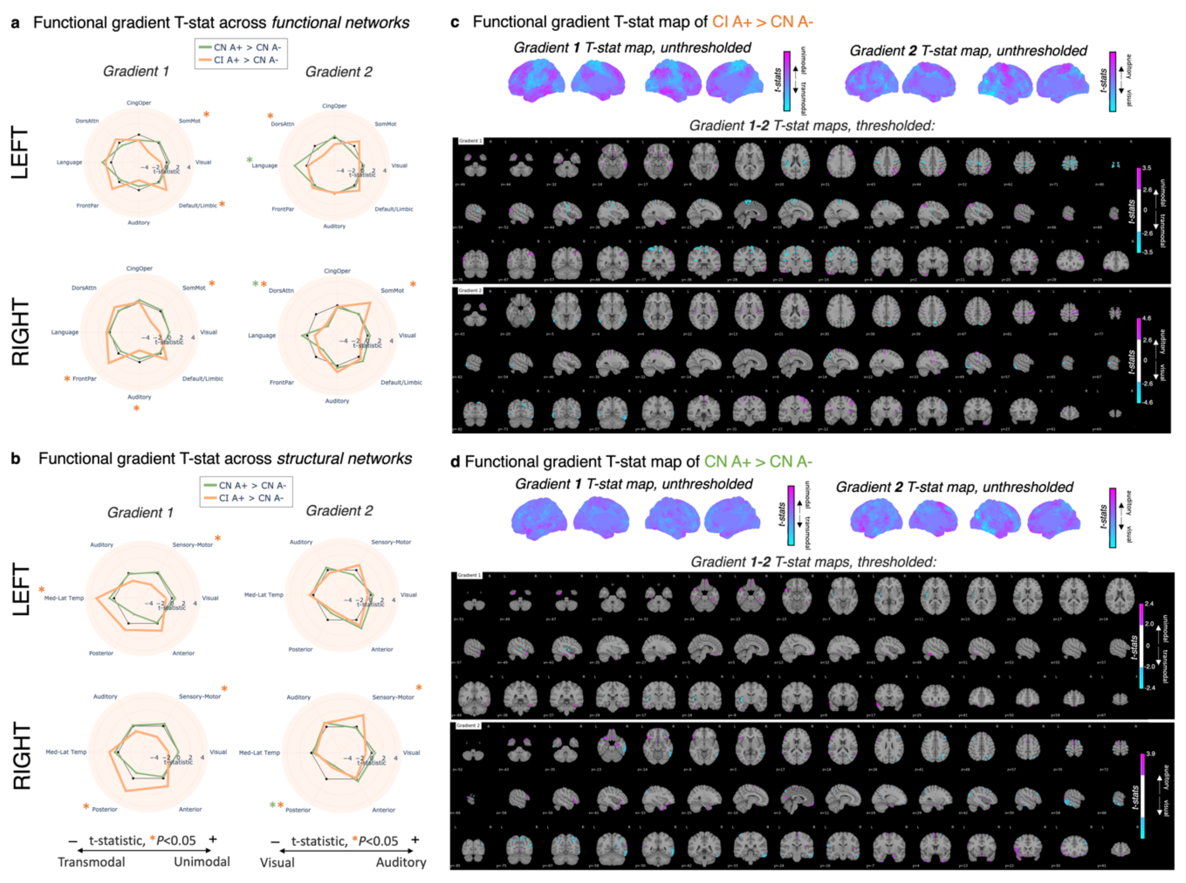

Supplementary Figure 5. Group-wise differences in <u>functional</u> gradient scores using an unbiased cohort-level realignment strategy. Panels **a-b**: Spider plots of the group-wise t-statistic differences in network-based gradient scores derived from the individual functional connectomes, using functional (Schaefer) networks or structural (Glasser) networks, respectively. Green and orange lines indicate CN A+ [n=35] > controls [n=101] and CI [n=72] > controls [n=101], respectively. The direction of the positive and negative t-statistic on the gradient is indicated at the bottom of the figure (e.g., regions with a negative t-statistic on $G1_{FC}$ moved closer towards the transmodal cortex in disease compared to controls). Asterisks represent significant t-statistics at the network-level with two-sided P<0.05, adjusted for age, sex, and *APOE*-ε4. Source data are provided as a Source Data file. Panels **c-d**: Group-wise t-statistic differences in gradient scores derived from the individual functional connectomes (c: CI > controls; d: CN A+ > controls). The direction of the positive and negative t-statistic on the gradient is indicated at the right side of the figure. T-statistics were adjusted for age, sex, and *APOE*-ε4, and family-wise errors due to multiple comparisons with a false-positive rate at two-sided P<0.01 and cluster-wise threshold of 500 voxels.

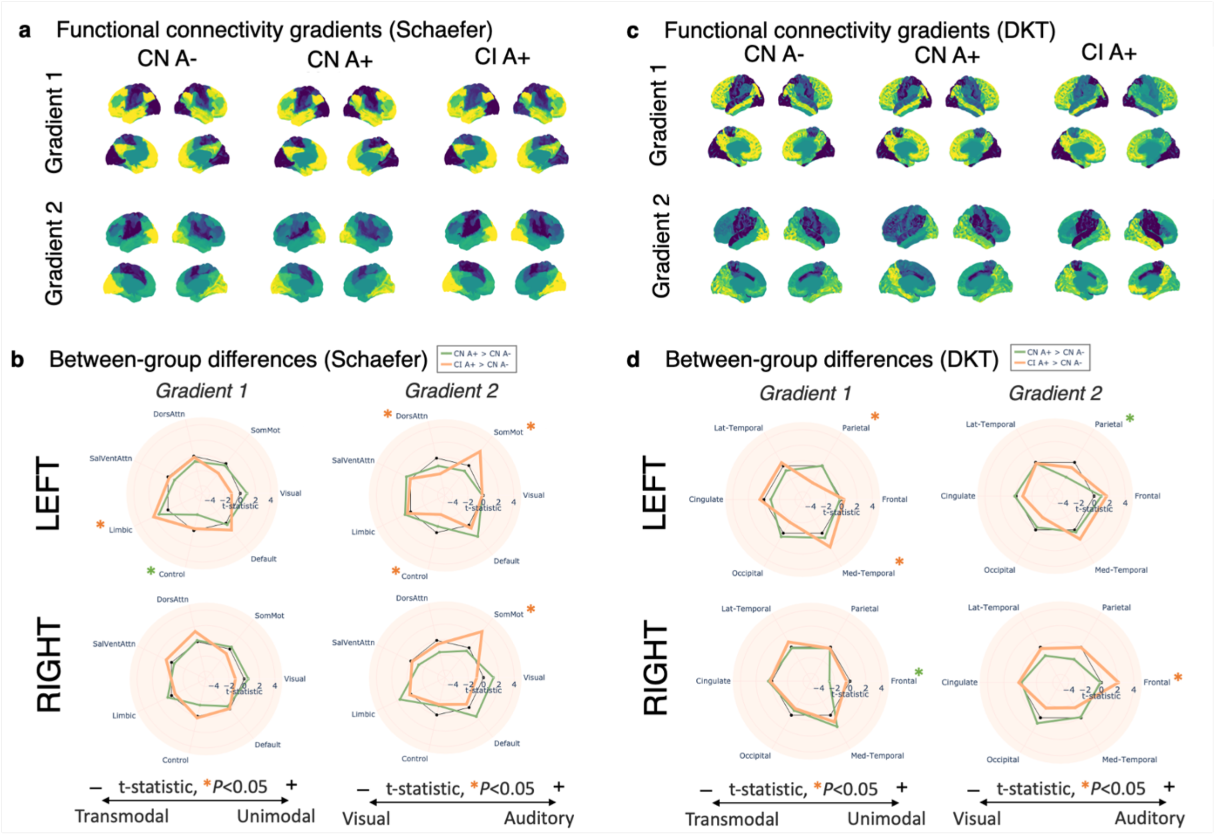

Supplementary Figure 6. <u>Functional</u> gradients derived from alternative (low-resolution) brain atlases. **a,** Gradient patterns per diagnostic group based on the Schaefer parcellation scheme (102 ROIs). **b,** Group-wise t-statistic differences in network-based gradient scores derived from the individual functional connectomes aligned to the cohort-level gradient template, using functional (Schaefer) ROIs averaged across 7 'Yeo' networks. Asterisks represent significant t-statistics at the network-level with two-sided P<0.05, adjusted for age, sex, and *APOE*-ε4. Panels **c** and **d** use the DKT parcellations (66 ROIs) and were averaged across structural (lobar) networks. Source data are provided as a Source Data file.

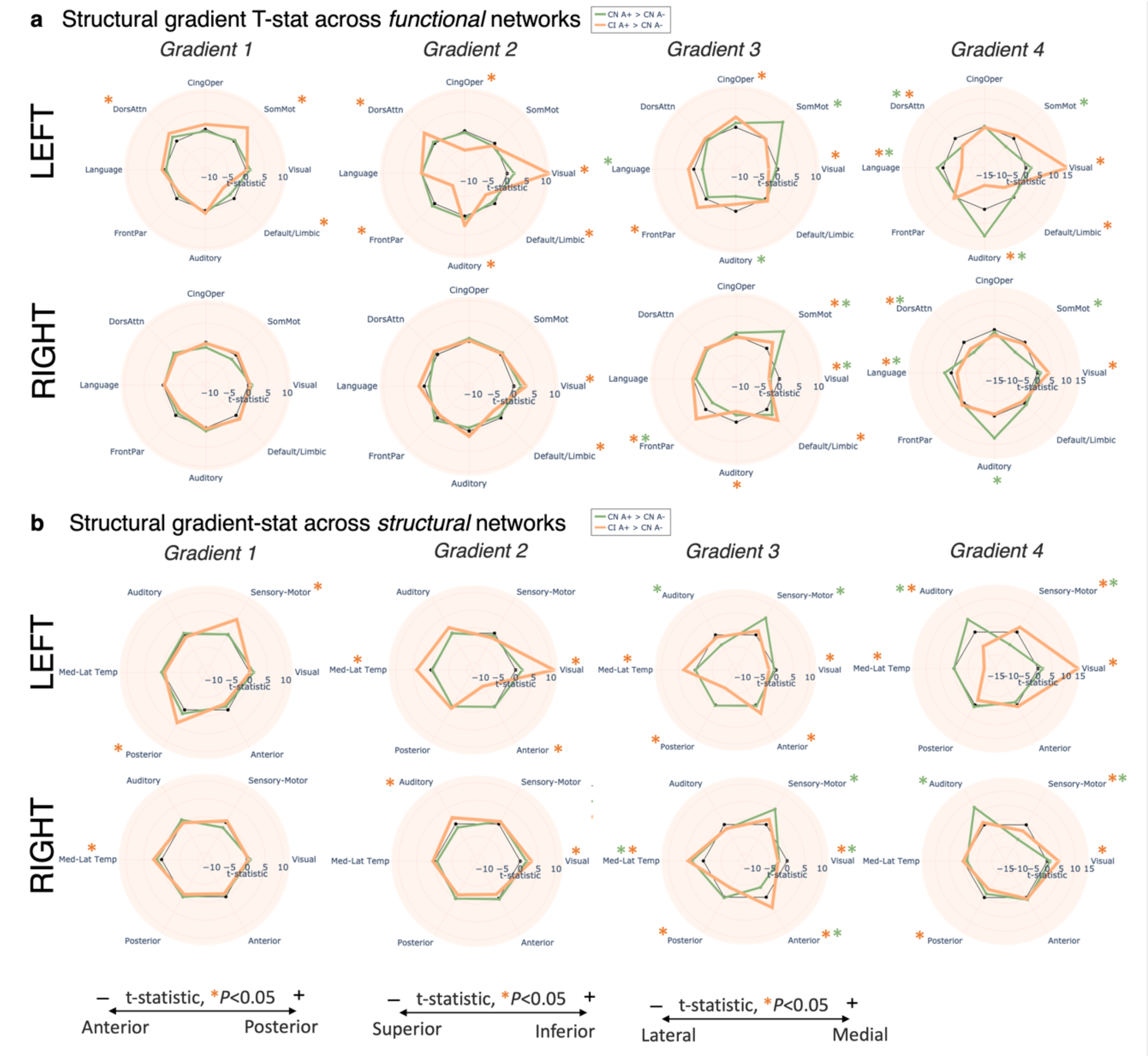

Supplementary Figure 7. Group-wise differences in network-based <u>structural</u> gradient scores aligned to group-wise gradient templates. Spider plots of the group-wise t-statistic differences in network-based gradient scores derived from the individual structural connectomes aligned to group-wise gradient templates. Panels **a** and **b** show the spider plots based on functional (Schaefer) networks and structural (Glasser) networks, respectively. Green and orange lines indicate CN A+ [n=35] > controls [n=102] and CI [n=72] > controls [n=102], respectively. The direction of the positive and negative t-statistic on the gradient is indicated at the bottom of the figure (e.g., regions with a negative t-statistic on $G1_{SC}$ moved closer towards the anterior cortex in disease compared to controls). Asterisks represent significant t-statistics at the network-level with two-sided P<0.05, adjusted for age, sex, and *APOE*-ε4. Source data are provided as a Source Data file.

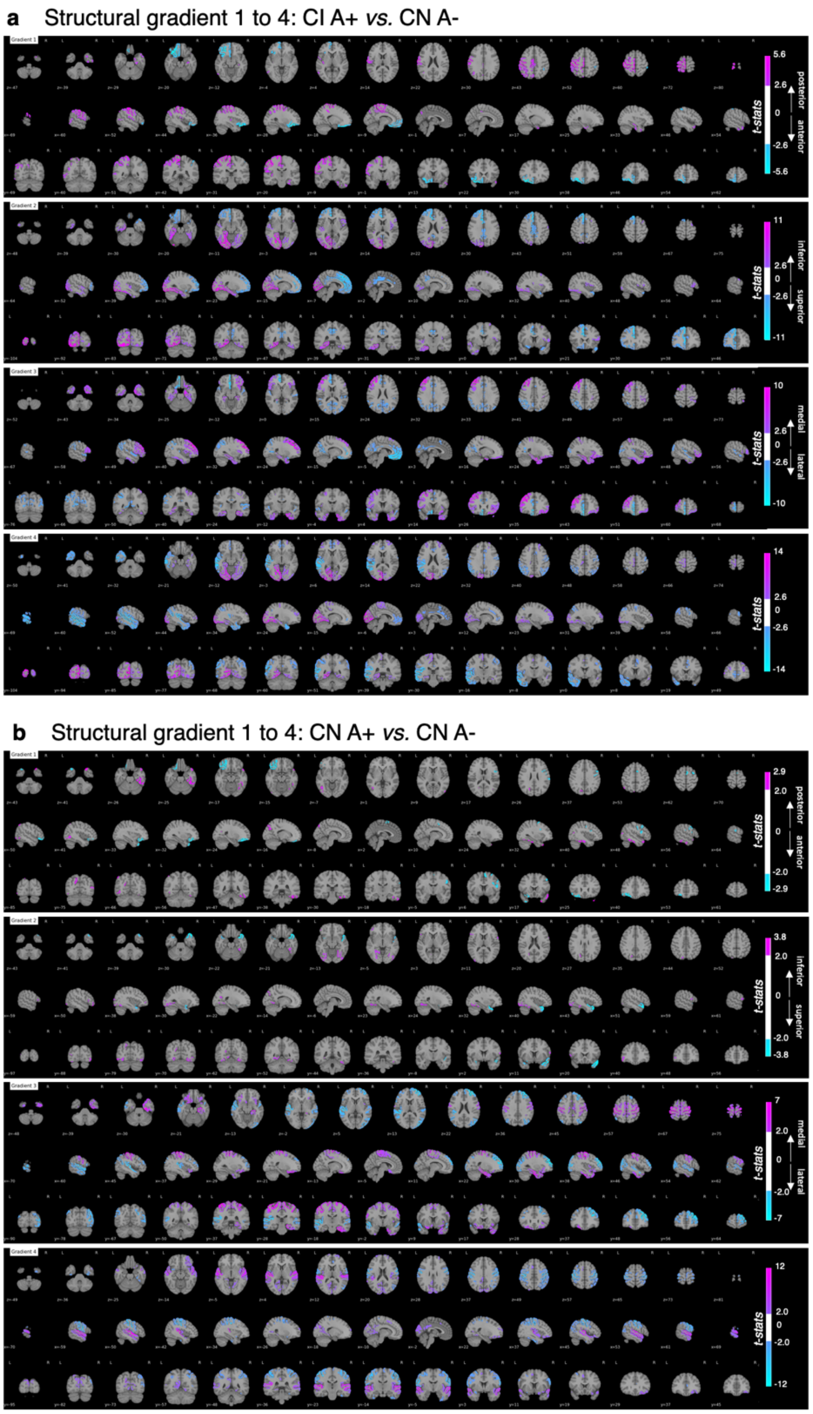

Supplementary Figure 8. Group-wise differences in brain-wide <u>structural</u> gradient scores aligned to group-wise gradient templates. Group-wise t-statistic differences in gradient scores derived from the individual structural connectomes aligned to group-wise gradient templates. Panels **a** and **b** show CI [n=72] > controls [n=102] and CN A+ [n=35] > controls [n=102], respectively. The direction of the positive and negative t-statistic on the gradient is indicated at the right side of the figure. T-statistics were adjusted for age, sex, and *APOE*-ε4, and family-wise errors due to multiple comparisons with a false-positive rate at two-sided P<0.01 and cluster-wise threshold of 500 voxels.

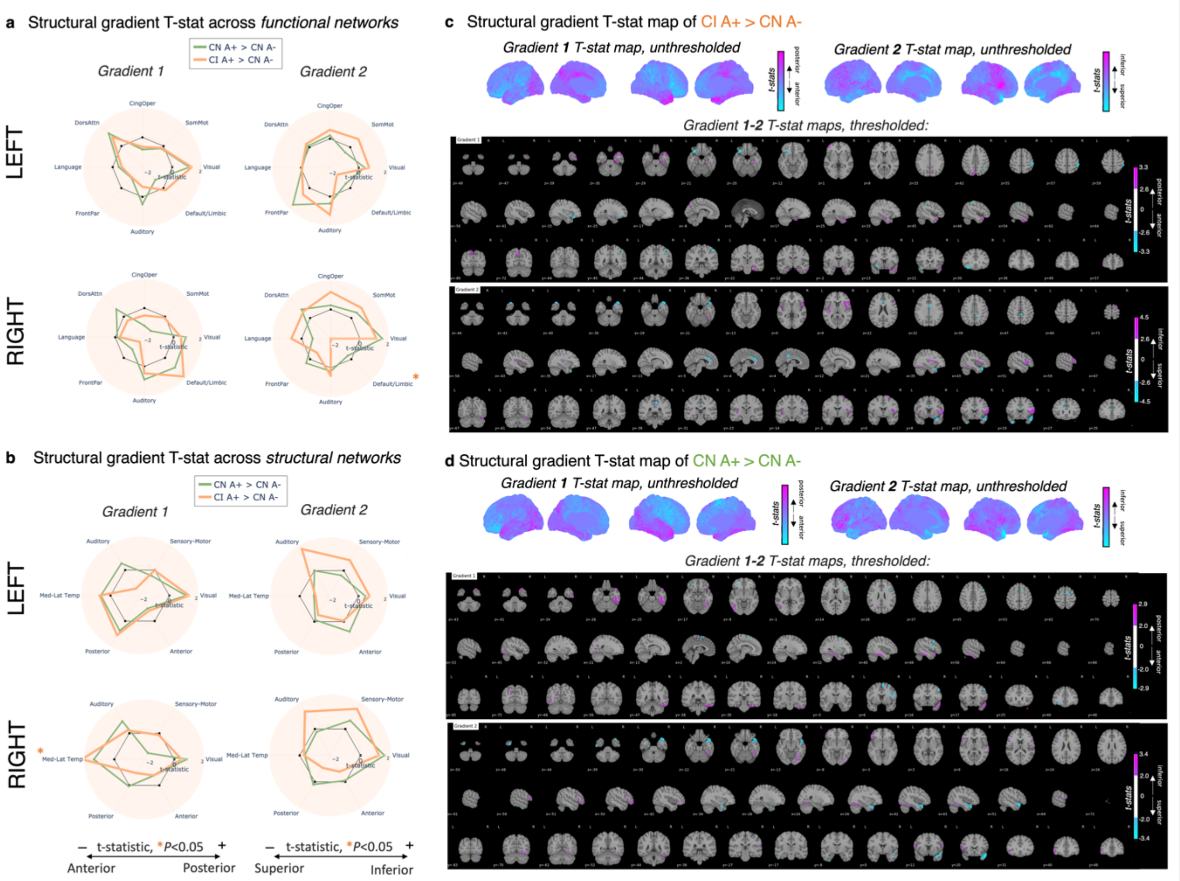

Supplementary Figure 9. Group-wise differences in <u>structural</u> gradient scores using an unbiased cohort-level realignment strategy. Panels **a-b**: Spider plots of the group-wise t-statistic differences in network-based gradient scores derived from the individual structural connectomes, using functional (Schaefer) networks or structural (Glasser) networks, respectively. Green and orange lines indicate CN A+ [n=35] > controls [n=102] and CI [n=72] > controls [n=102], respectively. The direction of the positive and negative t-statistic on the gradient is indicated at the bottom of the figure (e.g., regions with a negative t-statistic on $G1_{SC}$ moved closer towards the anterior cortex in disease compared to controls). Asterisks represent significant t-statistics at the network-level with two-sided P<0.05, adjusted for age, sex, and *APOE*-ε4. Source data are provided as a Source Data file. Panels **c-d**: Group-wise t-statistic differences in gradient scores derived from the individual structural connectomes (c: CI > controls; d: CN A+ > controls). The direction of the positive and negative t-statistic on the gradient is indicated at the right side of the figure. T-statistics were adjusted for age, sex, and *APOE*-ε4, and family-wise errors due to multiple comparisons with a false-positive rate at two-sided P<0.01 and cluster-wise threshold of 500 voxels.

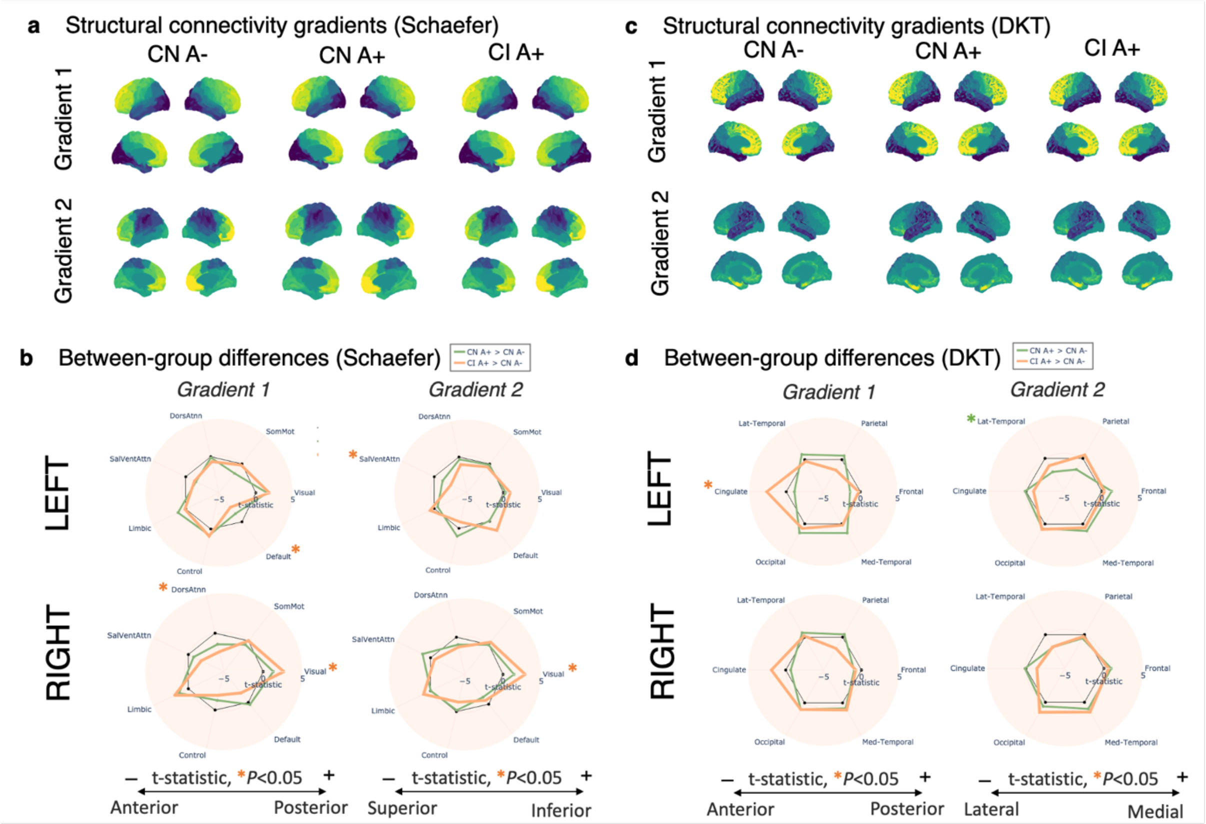

Supplementary Figure 10. **<u>Structural</u> gradients derived from alternative (low-resolution) brain atlases**. **a,** Gradient patterns per diagnostic group based on the Schaefer parcellation scheme (102 ROIs). **b,** Group-wise t-statistic differences in network-based gradient scores derived from the individual structural connectomes aligned to the cohort-level gradient template, using functional (Schaefer) ROIs averaged across functional 7 'Yeo' networks. Asterisks represent significant t-statistics at the network-level with two-sided P<0.05, adjusted for age, sex, and *APOE*-ε4. Panels **c** and **d** use the DKT parcellations (66 ROIs) and were averaged across structural (lobar) networks. Source data are provided as a Source Data file.

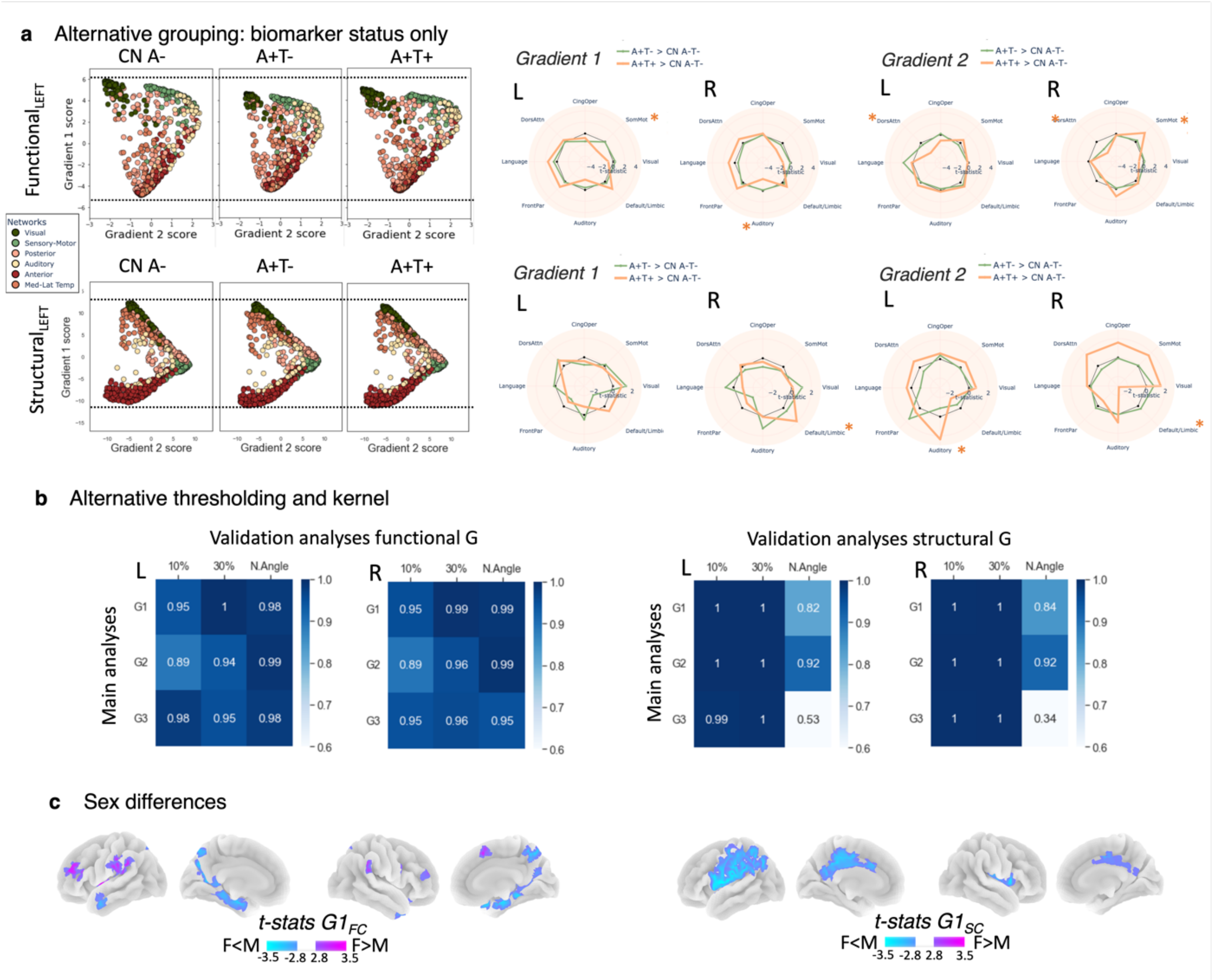

Supplementary Figure 11. Sensitivity analyses of the connectome gradients. **a,** Gradient extraction and group-wise comparison using biomarker-based diagnostic groups (A-T-, A+T-, A+T+). Gradients derived from the group-wise connectome (left panel); group-wise t-statistic differences in network-based gradient scores derived from the individual connectomes aligned to the cohort-level gradient template (right panel). **b,** Spatial correlation coefficients (Pearson's R) between G_{FC} (left panel) or G_{SC} (right panel) in the main analysis and those in the validation analyses (sparsity thresholding at 10% and 30%, and normalized angle kernel). **c**, Sex effects (FWE-corrected at two-sided P<0.005). M male, F female. Source data are provided as a Source Data file.

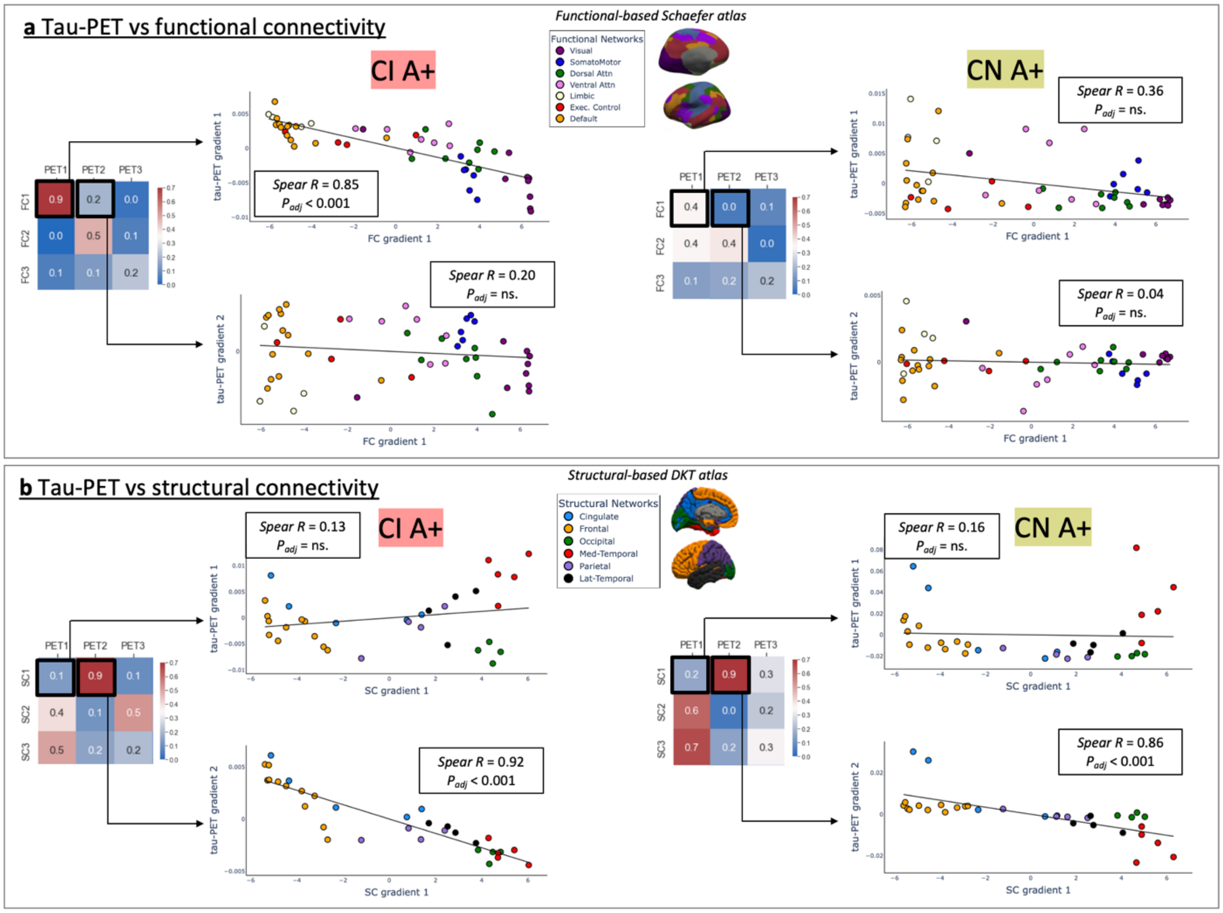

Supplementary Figure 12. Connectivity vs. tau-PET gradients. Spearman's rank correlations between G_{TAU} and G_{FC} (panel **a**) or G_{SC} (panel **b**) for the left hemisphere in CI (left panel, n=75) and CN A+ (right panel, n=35). Datapoints correspond to regions, which were extracted from the functional-based Schaefer atlas or the structural-based DKT atlas. The two-sided P-value of gradient correlations was tested with null models using spatial autocorrelation-preserving surrogates based on variogram matching (1000 permutations). Source data are provided as a Source Data file.

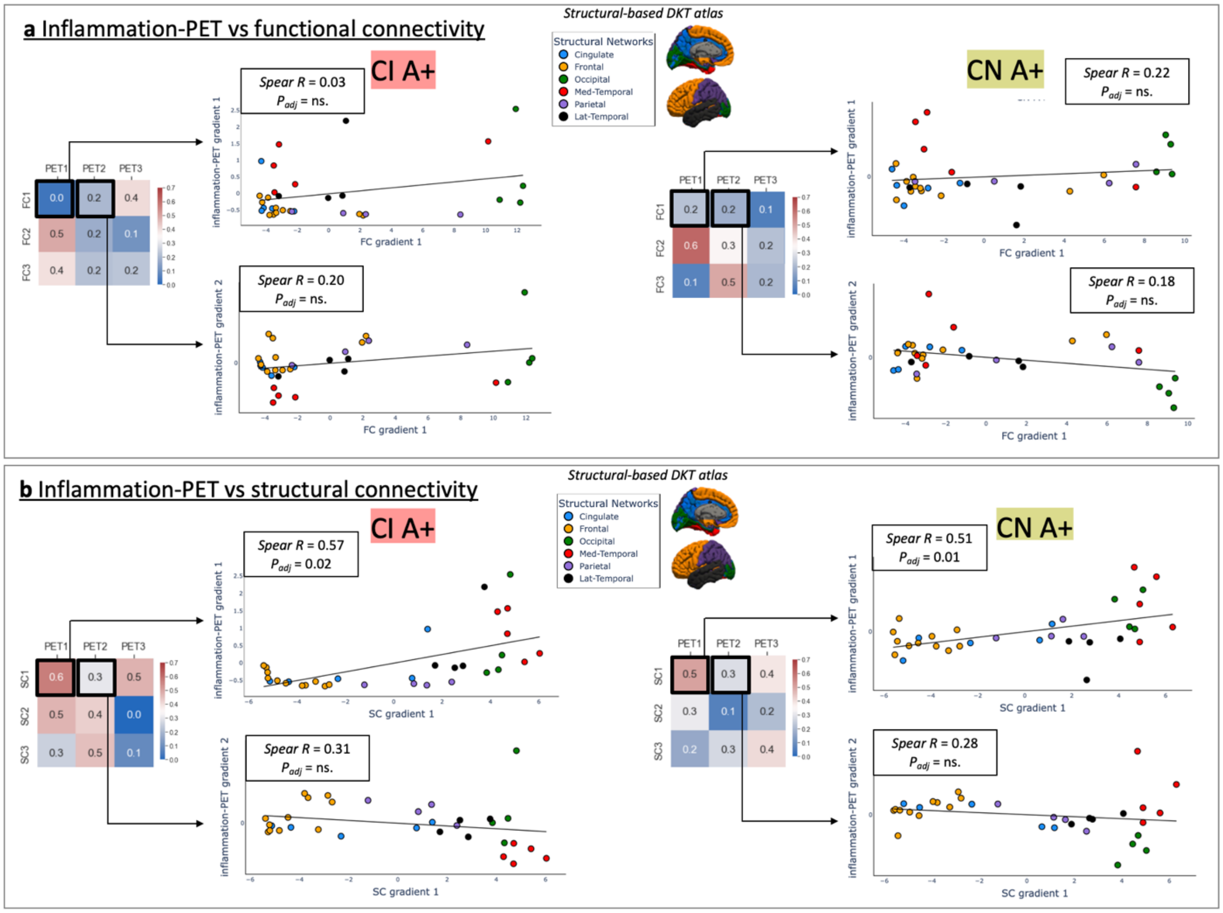

Supplementary Figure 13. Connectivity vs. inflammation-PET gradients. Spearman's rank correlations between G_{INFLAM} and G_{FC} (panel **a**) or G_{SC} (panel **b**) for the left hemisphere in CI (left panel, n=32) and CN A+ (right panel, n=18). Datapoints correspond to regions, which were extracted from the structural-based DKT atlas (the functional-based Schaefer atlas sub-performed in terms of reliable gradient extraction for TSPO-PET). The two-sided P-value of gradient correlations was tested with null models using spatial autocorrelation-preserving surrogates based on variogram matching (1000 permutations). Source data are provided as a Source Data file.

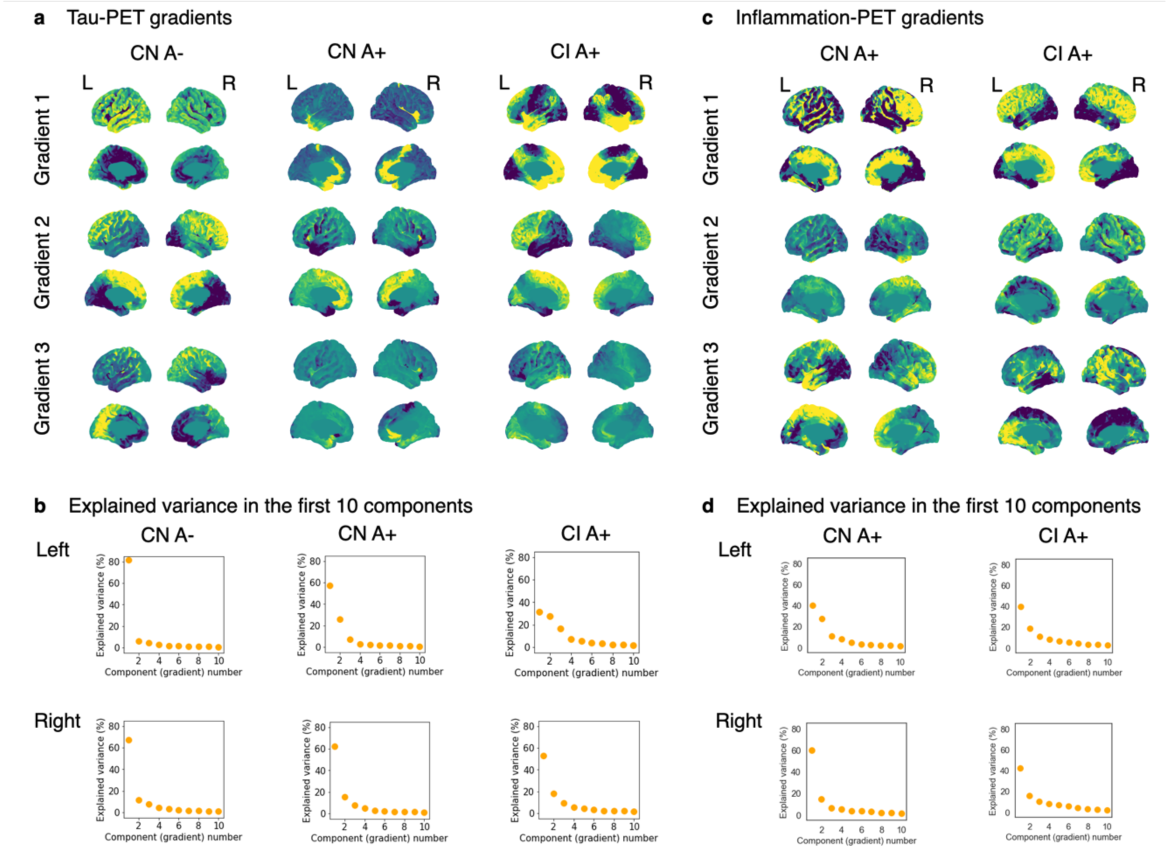

Supplementary Figure 14. PET gradient patterns across diagnostic groups. Panels **a** and **c** show the first three G_{TAU} and G_{INFLAM}, respectively, derived from the group-averaged connectomes. The CN connectomes (n=103 controls and n=35 CN A+) were thresholded at 50% sparsity for tau-PET and the CN (n=18) and CI (n=32) connectomes were thresholded at 50% for inflammation-PET. Validation of thresholding is further shown in Supplementary Figure 16. Source data are provided as a Source Data file. Panels **b** and **d** show the explained information in the PET data across the first ten cohort-level gradients for the left and right hemisphere.

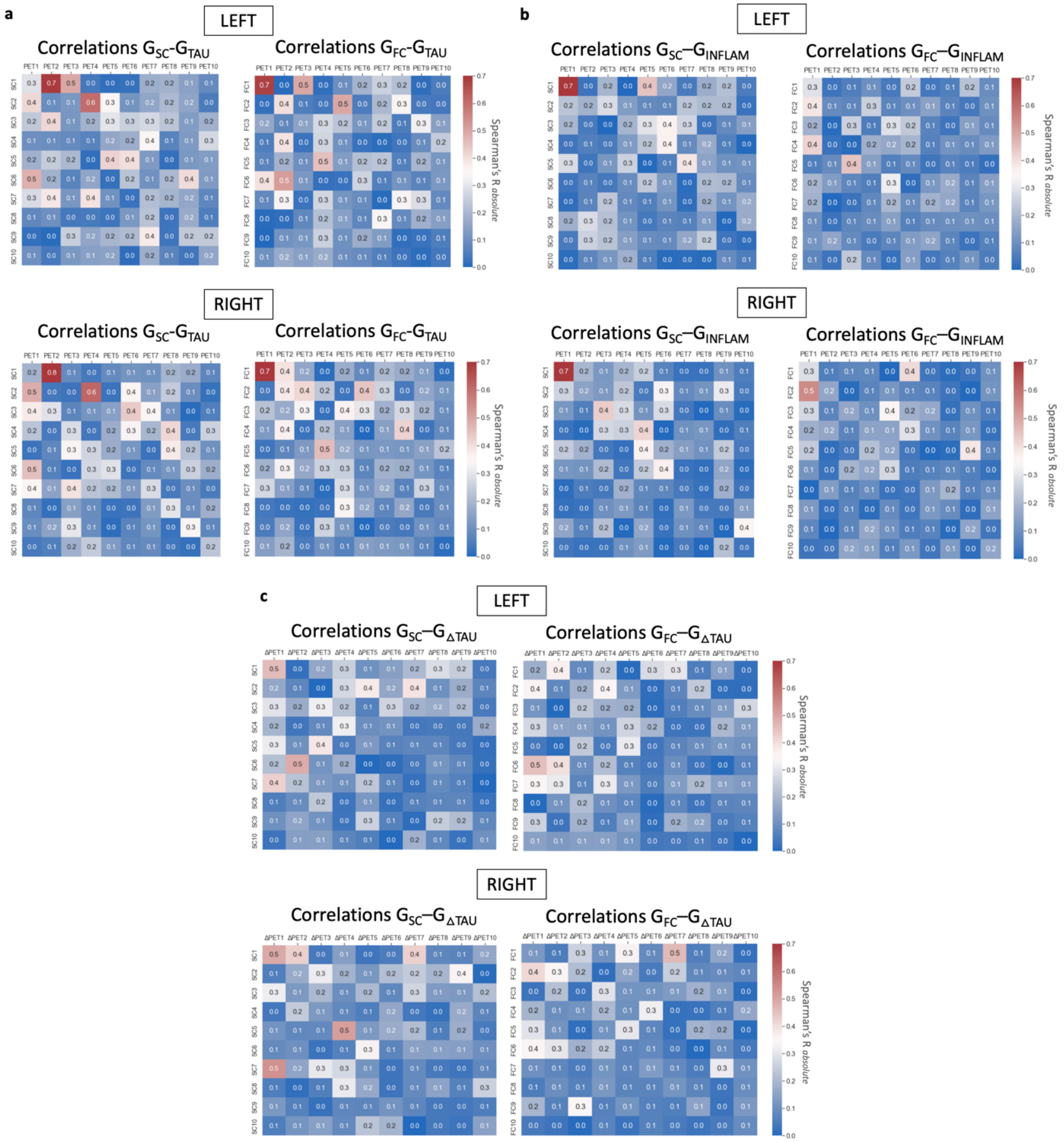

Supplementary Figure 15. PET-connectome gradient correlations among the first 10 components. **a,** Heatmap showing Spearman's rank correlations between tau-PET and connectome gradients in CI (n=75); **b,** Heatmap showing Spearman's rank correlations between inflammation-PET and connectome gradients in CI (n=32); **c** heatmap showing Spearman's rank correlations between Δtau-PET and connectome gradients in all A+ combined (n=39).

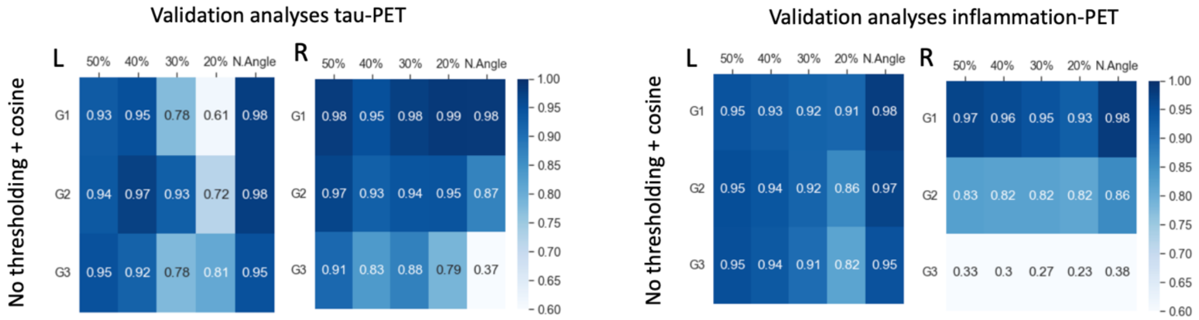

Supplementary Figure 16. Sensitivity analyses of the PET gradients. Spatial correlation coefficients (Pearson's R) between tau (left panel) or inflammation (right panel) PET gradients in the main (no thresholding + cosine similarity kernel) analyses and those in the validation analyses.

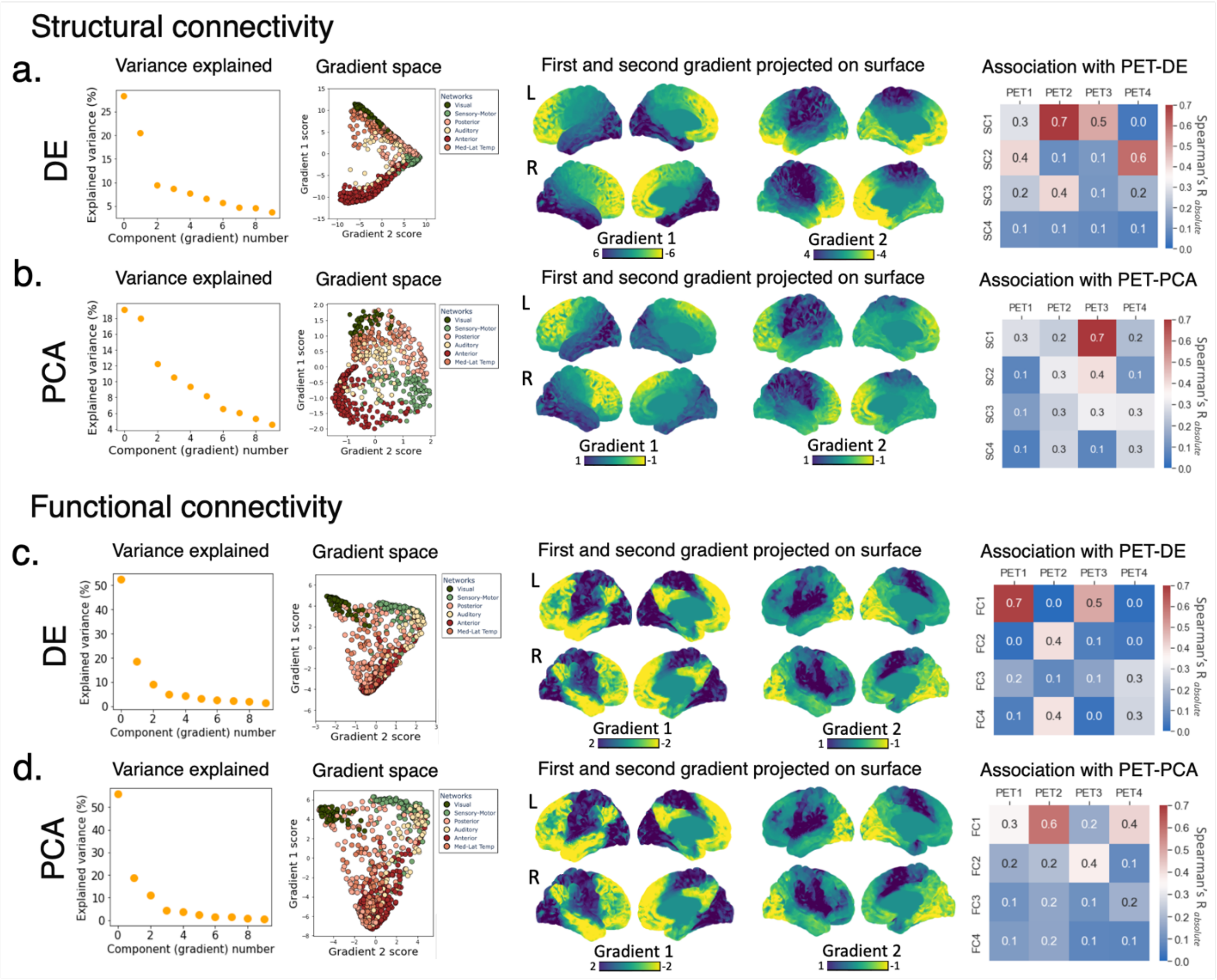

Supplementary Figure 17. Comparison between diffusion embedding (DE) and principal component analysis (PCA) for dimensionality reduction. Left to right: Information explained in the first 10 components after decomposition of the structural affinity matrix, indicating higher information explained in the components of DE (**panel a**; component 1 and 2: 23 and 20%) compared to PCA (**panel b**; component 1 and 2: 19 and 18%); gradient space based on the first two components; first two components projected on the surface; correlation matrix showing the association between connectome and PET components, indicating more significant associations for DE. Results are shown for the CI group. **c-d,** Results for functional connectivity. Source data are provided as a Source Data file.

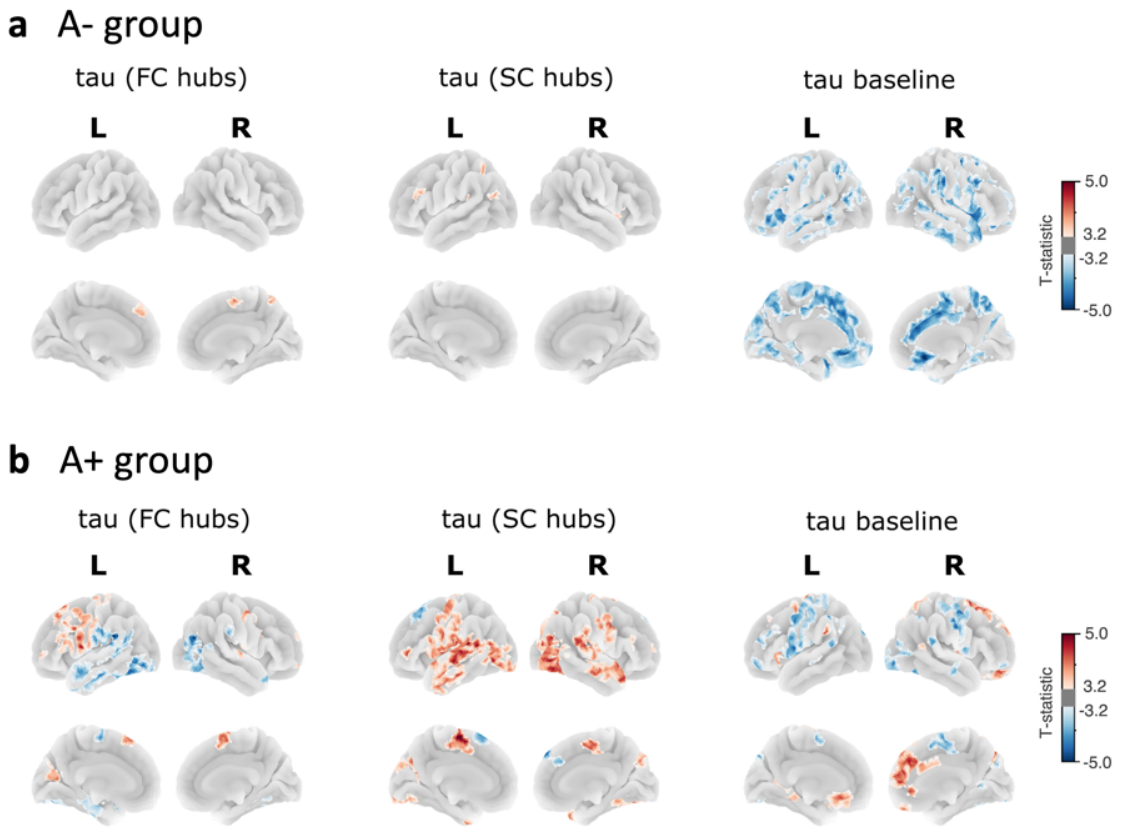

Supplementary Figure 18. Tau within gradient-derived subject-specific hubs drives tau accumulation within the A+ group. Tau accumulation over time (ΔSUVR) within an ROI is predicted by baseline tau SUVR within gradient-derived functional hubs connected to the ROI (first column), as well as by baseline tau SUVR within gradient-derived structural hubs connected to the ROI (middle column), and by baseline tau SUVR within the ROI (right column), in A+ participants (n=39, panel **b**) but not A- participants (n=47, panel **a**). A positive t-statistic (red) within an ROI indicated a positive relationship between tau accumulation within the ROI and baseline tau within the ROI's subject-specific hubs. Linear regression adjusted for age, sex, *APOE*-ε4, baseline tau and FWE at two-sided p<0.001. Source data are provided as a Source Data file.

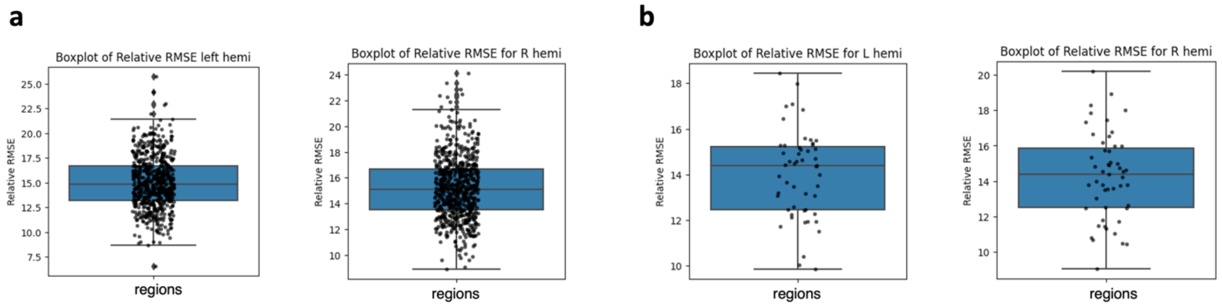

Supplementary Figure 19. Leave-one-out cross-validation results of the association between longitudinal tau accumulation and baseline tau within gradient-derived subject-specific hubs. The y-axis represents the relative root-mean-squared-error (Relative RMSE in %) for each of the regional analyses, indicating good model performance. **a,** High-resolution atlas for left and right hemisphere. **b,** Low-resolution (Schaefer) atlas for left and right hemisphere.

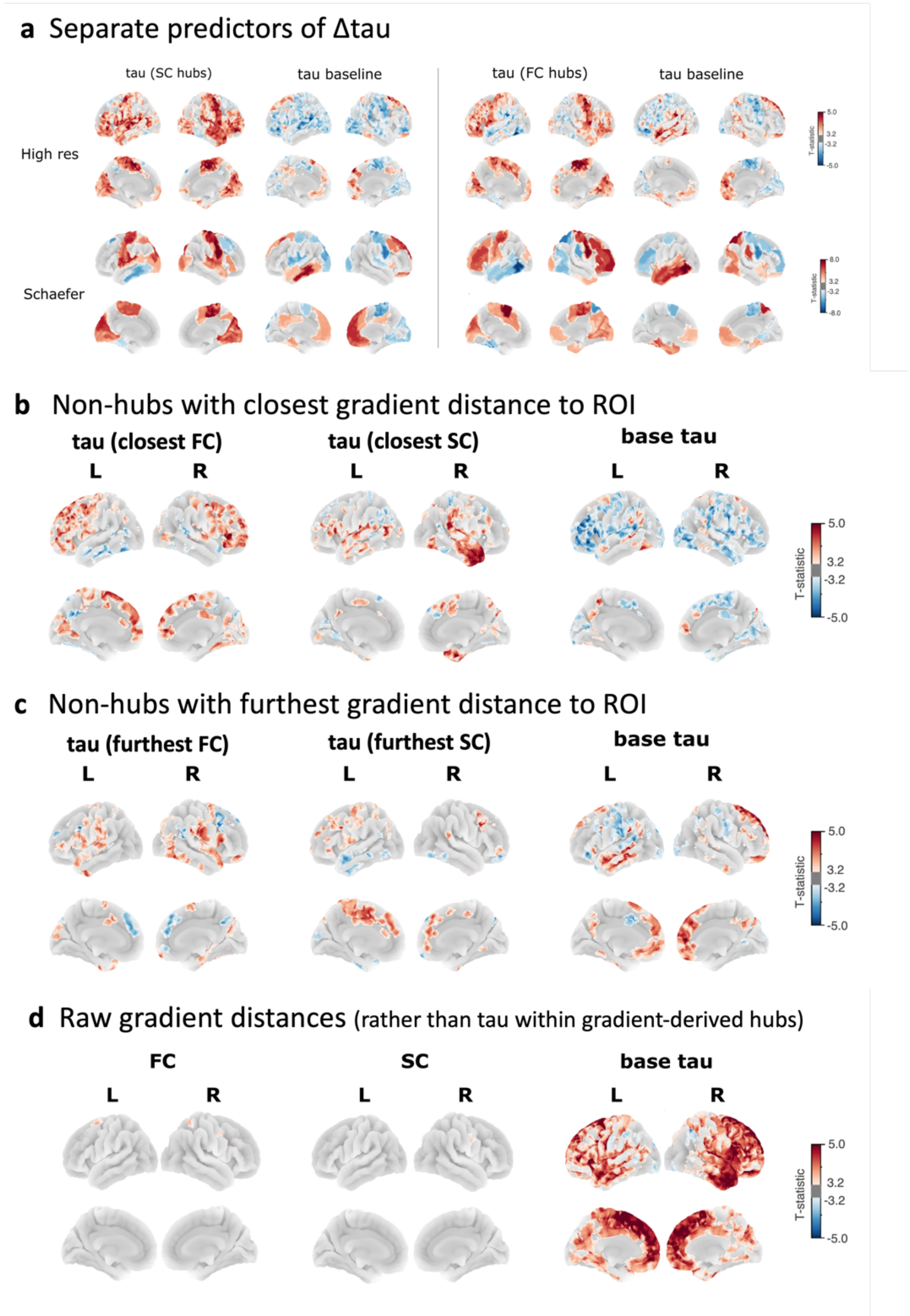

Supplementary Figure 20. Sensitivity analyses of tau accumulation prediction using gradients. **a,** Using tau within FC or SC hubs as separate predictors in the main model (n=86 participants). Source data are provided as a Source Data file. **b,** When quantifying tau within the nodes with strongest gradient-based connectivity to each ROI (that is, shortest Euclidean distance in gradient space), we observed that higher tau accumulation in ROI was facilitated by higher tau deposition within the ROI's closest connections, irrespective of hub status. **c,** When selecting non-hubs with weak gradient-based connectivity to the ROI, effect sizes were weaker. **d,** When repeating our analysis using raw FC and SC gradient distances as predictors of tau accumulation (rather than tau within these connections), only higher baseline tau (but not FC or SC) predicted faster tau progression.

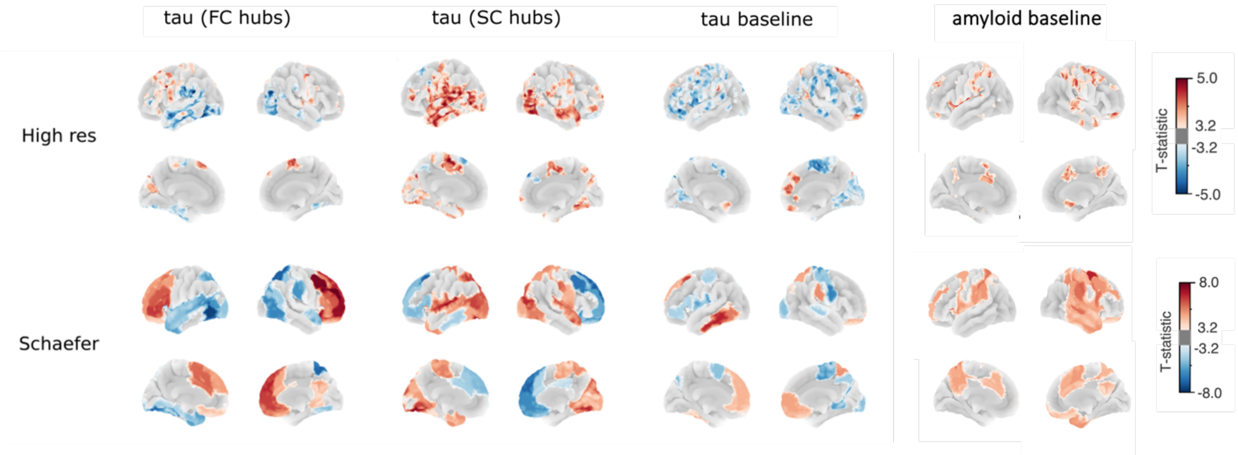

Supplementary Figure 21. Adjustment for amyloid. Tau accumulation over time (ΔSUVR) within an ROI is predicted by baseline tau SUVR within gradient-derived functional hubs connected to the ROI (first column), as well as by baseline tau levels within gradient-derived structural hubs connected to the ROI (second column), and by baseline ROI tau (third column) and amyloid (last column) (n=86 participants). A positive t-statistic (red) within an ROI indicated a positive relationship between tau accumulation within the ROI and baseline tau or amyloid within the ROI's subject-specific hubs. Linear regression adjusted for age, sex, *APOE*-ε4, baseline tau and amyloid and FWE at two-sided $p<0.001$, replicated for two different atlases. Source data are provided as a Source Data file.

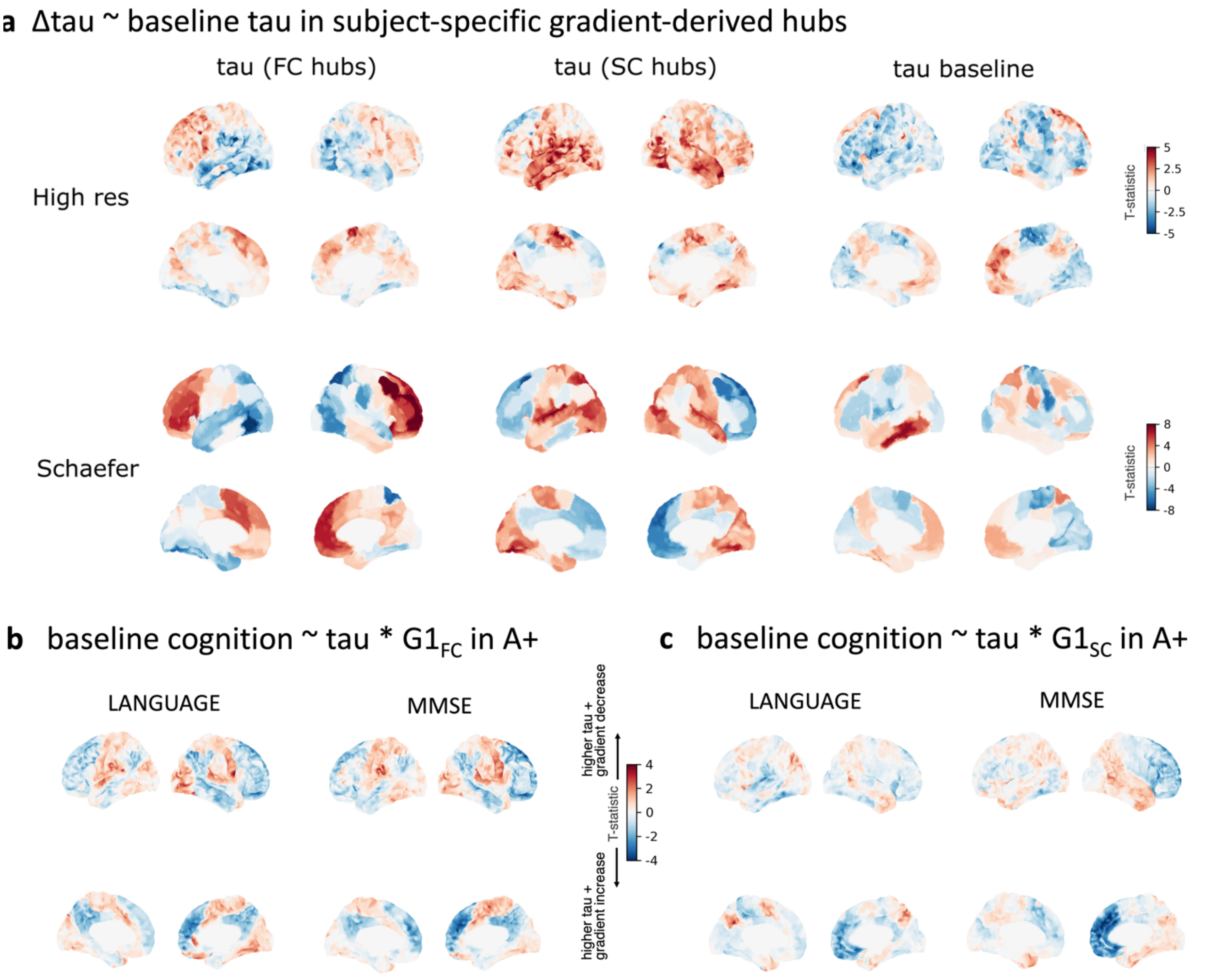

Supplementary Figure 22. Unthresholded t-stat maps to observe overall trends. a, Tau accumulation over time (ΔSUVR) within an ROI is predicted by baseline tau SUVR within gradient-derived functional hubs connected to the ROI (first column), as well as by baseline tau SUVR within gradient-derived structural hubs connected to the ROI (middle column), and by baseline tau SUVR within the ROI (right column) (n=86 participants). **b,** The interaction effect between regional tau SUVR and $G1_{FC}$ on cognition in A+ (MMSE n=107 and language n=90 participants). A negative interaction effect (blue t-statistic in transmodal regions) indicates that higher tau together with higher $G1_{FC}$ score (gradient contraction towards unimodal) results in lower cognition. Similarly, a positive interaction (red t-statistic in unimodal regions) indicates that higher tau together with lower $G1_{FC}$ score (gradient contraction towards transmodal) is associated with lower cognition. **c,** The interaction between regional tau SUVR and $G1_{SC}$ on cognition.

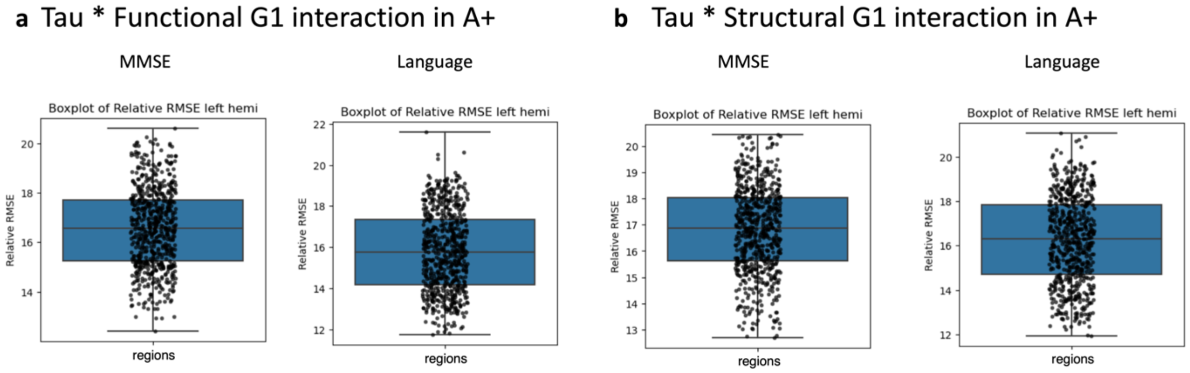

Supplementary Figure 23. Leave-one-out cross-validation results for the interaction between tau and G1 on cognition. **a,** Interaction effect between regional tau SUVR and $G1_{FC}$ on cognition. **b,** Interaction between regional tau SUVR and $G1_{SC}$ on cognition. The primary cognitive domains included MMSE and language. The y-axis represents the relative root-mean-squared-error (Relative RMSE in %) for each of the regional interaction analyses, indicating good model performance. Results are shown for the left hemisphere (with similar results for the right side).

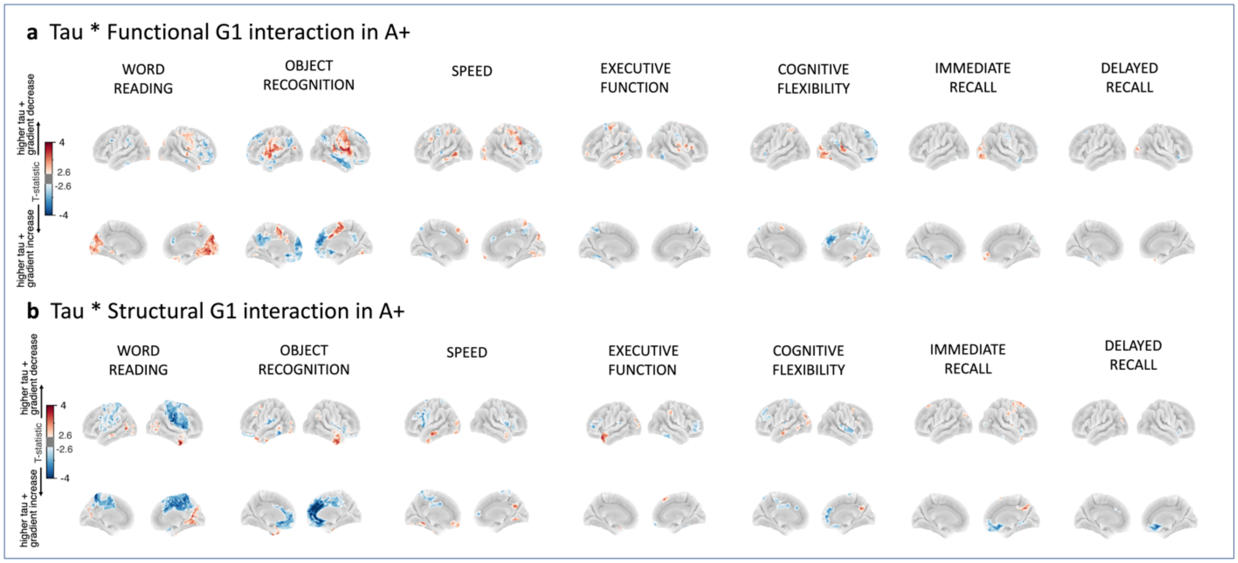

Supplementary Figure 24. Interaction effects between connectome gradients and tau on cognition in A+. a, Interaction effect between regional tau SUVR and $G1_{FC}$ on cognition. **b,** Interaction between regional tau SUVR and $G1_{SC}$ on cognition. All analyses were adjusted for age, sex, education, *APOE*-ε4 and FWE at two-sided p<0.01. Sample sizes of A+ varied across composite scores: word reading n=87, delayed memory n=76, immediate memory n=82, executive function n=86, object recognition n=86, processing speed n=84, and cognitive flexibility n=81.

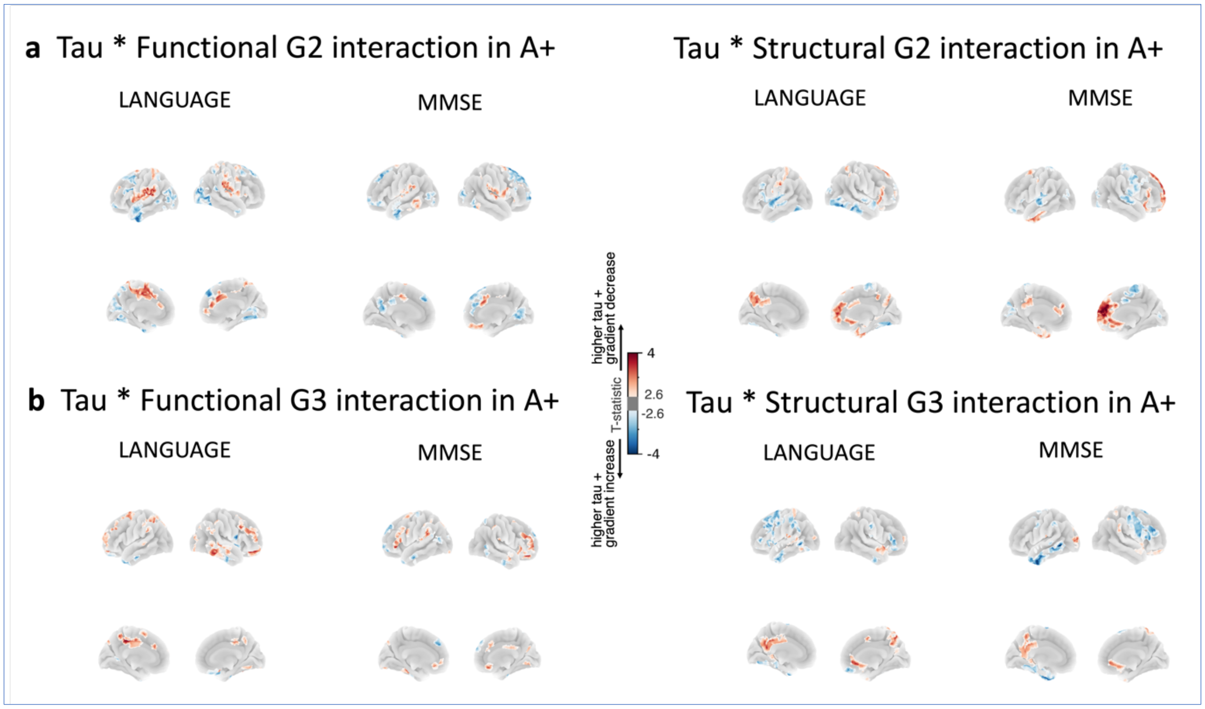

Supplementary Figure 25. Interaction effects between higher-order connectome gradients and tau on cognition in A+. a, Interaction effect between regional tau SUVR and G2 on cognition in A+ (MMSE n=107 and language n=90 participants). **b,** Analysis repeated with G3 score. All analyses were adjusted for age, sex, education, *APOE*-ε4 and FWE at two-sided p<0.01.

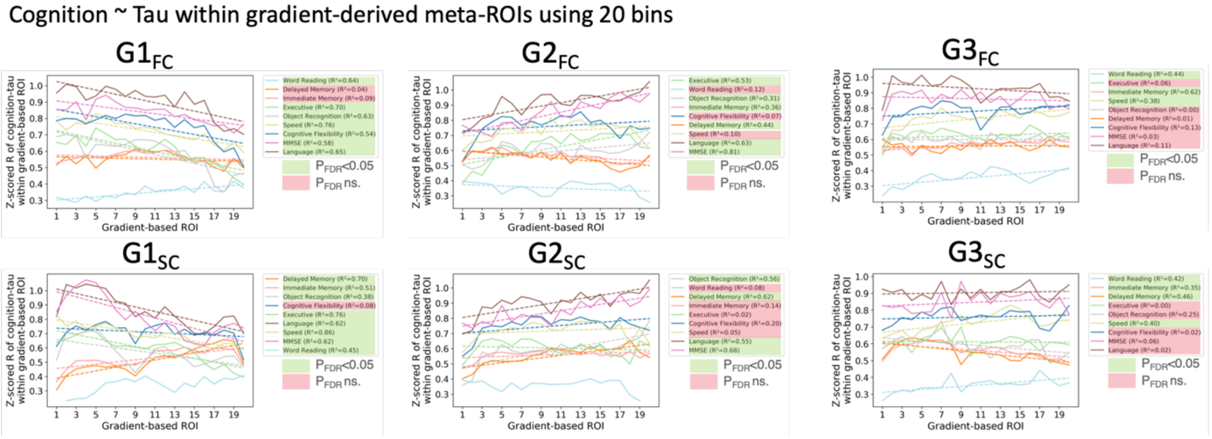

Supplementary Figure 26. Gradual transition of the tau-cognition relationship along the highest-variance explaining gradient axes of connectivity. The Y-axis represents the [z-scored] partial correlation coefficient R (adjusted for age, sex, education, and *APOE*-ε4) between cognitive score and tau averaged within consecutive, equally-sized meta-ROIs along the connectome gradient; the X-axis corresponds to the meta-ROI bin number. Cognitive domains for which the linear fit between z-scored R and bin number survived FDR correction are highlighted in green, indicating a gradual or 'progressive' transition of the tau-cognition relation along the principal axes of brain organization at two-sided $P_{FDR}<0.05$. Sample sizes of A+ participants varied across composite scores: word reading n=87, delayed memory n=76, immediate memory n=82, executive function n=86, object recognition n=86, processing speed n=84, and cognitive flexibility n=81. Source data are provided as a Source Data file.

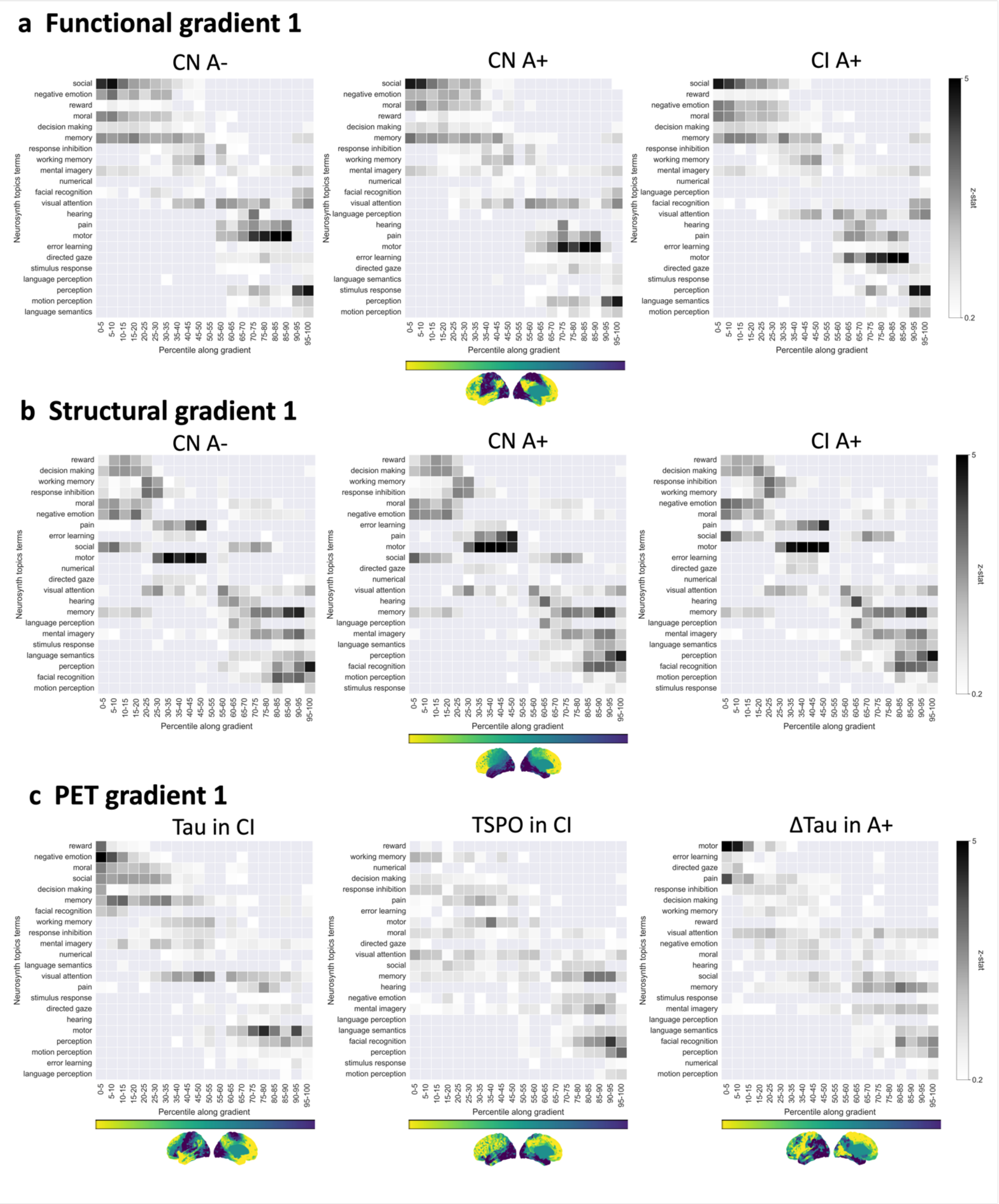

Supplementary Figure 27. Associations between meta-analytic cognitive terms and primary gradients. Cognitive terms are derived from the NeuroSynth database and ordered by the weighted mean of their location along 5-percentile bins of our primary template gradients (a: functional gradient, b: structural gradient, c: PET gradients) (Margulies et al. 2016 PNAS). Source data are provided as a Source Data file.

Supplementary Table 1. Goodness-of-fit metrics corresponding to main Figure 3.

	Tanh	Cubic	Linear
	RMSE, R^2	RMSE, R^2	RMSE, R^2
$G1_{FC}$-$G1_{TAU}$	0.0047, 0.41	**0.0044, 0.50**	0.0047, 0.41
$G1_{SC}$-$G2_{TAU}$	0.0028, 0.57	**0.0029, 0.56**	0.0034, 0.39
$G2_{FC}$-$G1_{INFLAM}$	0.2082, 0.24	**0.1883, 0.38**	0.2082, 0.24
$G1_{SC}$-$G1_{INFLAM}$	0.1801, 0.43	**0.1683, 0.50**	0.1801, 0.43

Supplementary Table 2. Goodness-of-fit metrics corresponding to main Figure 4.

	Tanh	Cubic	Linear
	RMSE, R^2	RMSE, R^2	RMSE, R^2
$G1_{FC}$-$G2_{\Delta TAU}$	0.0420, 0.13	**0.0387, 0.26**	0.0420, 0.13
$G2_{FC}$-$G1_{\Delta TAU}$	0.1636, 0.12	**0.1635, 0.12**	0.1657, 0.10
$G1_{SC}$-$G1_{\Delta TAU}$	0.1448, 0.31	**0.1464, 0.30**	0.1567, 0.19
