# Supplementary material for: Tau follows principal axes of functional and structural brain organization in Alzheimer’s disease
Source: Nat Commun. 2024 Jun 12;15:5031. doi: 10.1038/s41467-024-49300-2 (PMC11169286; doi:10.1038/s41467-024-49300-2)
Supplement: Supplementary file 3 — Reporting Summary [file 41467_2024_49300_MOESM3_ESM.pdf]

nature portfolio

Corresponding author(s): Goubran, Maged

Last updated by author(s): Apr 1, 2024

Reporting Summary

Nature Portfolio wishes to improve the reproducibility of the work that we publish. This form provides structure for consistency and transparency in reporting. For further information on Nature Portfolio policies, see our Editorial Policies and the Editorial Policy Checklist.

Statistics

For all statistical analyses, confirm that the following items are present in the figure legend, table legend, main text, or Methods section.

n/a	Confirmed	
☐	☒	The exact sample size (*n*) for each experimental group/condition, given as a discrete number and unit of measurement
☐	☒	A statement on whether measurements were taken from distinct samples or whether the same sample was measured repeatedly
☐	☒	The statistical test(s) used AND whether they are one- or two-sided<br>*Only common tests should be described solely by name; describe more complex techniques in the Methods section.*
☐	☒	A description of all covariates tested
☐	☒	A description of any assumptions or corrections, such as tests of normality and adjustment for multiple comparisons
☐	☒	A full description of the statistical parameters including central tendency (e.g. means) or other basic estimates (e.g. regression coefficient) AND variation (e.g. standard deviation) or associated estimates of uncertainty (e.g. confidence intervals)
☐	☒	For null hypothesis testing, the test statistic (e.g. *F*, *t*, *r*) with confidence intervals, effect sizes, degrees of freedom and *P* value noted<br>*Give P values as exact values whenever suitable.*
☒	☐	For Bayesian analysis, information on the choice of priors and Markov chain Monte Carlo settings
☒	☐	For hierarchical and complex designs, identification of the appropriate level for tests and full reporting of outcomes
☐	☒	Estimates of effect sizes (e.g. Cohen's *d*, Pearson's *r*), indicating how they were calculated

Our web collection on statistics for biologists contains articles on many of the points above.

Software and code

Policy information about availability of computer code

Data collection	No software used to collect the data.
Data analysis	All statistical analyses were performed in Python v3.7.<br>Imaging software:<br>- ICVMapper v1 (https://icvmapp3r.readthedocs.io/en/latest/)<br>- FreeSurfer v7<br>- fMRIPrep v.20.2.3<br>- FSL v6.0.5, ANTS v2.3.1, MRtrix3 v3.0.3<br>Analysis software:<br>- Neurosynth (https://neurosynth.org/analyses/topics/v5-topics-50/)<br>- BrainSpace v0.1.3<br>Example code and simulated data for gradient extraction available via: https://github.com/AICONSlab/AD_connectome_gradients.

For manuscripts utilizing custom algorithms or software that are central to the research but not yet described in published literature, software must be made available to editors and reviewers. We strongly encourage code deposition in a community repository (e.g. GitHub). See the Nature Portfolio guidelines for submitting code & software for further information.

Data

Policy information about availability of data

All manuscripts must include a data availability statement. This statement should provide the following information, where applicable:

- Accession codes, unique identifiers, or web links for publicly available datasets
- A description of any restrictions on data availability
- For clinical datasets or third party data, please ensure that the statement adheres to our policy

All requests for raw and analyzed data and materials will be promptly reviewed by McGill University to verify if the request is subject to any intellectual property or confidentiality obligations. Anonymized data will be shared upon request to the study's senior author from a qualified academic investigator for sole the purpose of replicating the procedures and results presented in this article. Any data and materials that can be shared will be released via a material transfer agreement. Data are not publicly available due to information that could compromise the privacy of research participants. Related documents, including study protocol and informed consent forms, can similarly be made available upon request. NeuroSynth database with meta-analytic topic terms is available at https://neurosynth.org/analyses/topics/v5-topics-50/. Source data are provided with this paper.

Research involving human participants, their data, or biological material

Policy information about studies with human participants or human data. See also policy information about sex, gender (identity/presentation), and sexual orientation and race, ethnicity and racism.

Reporting on sex and gender	All analyses are adjusted for sex based on self-report (F=60.6%). Our dataset did not record gender at the time of data collection. Gradient effects disaggregated for sex (Supplementary Fig.11c)
Reporting on race, ethnicity, or other socially relevant groupings	All patients are White.
Population characteristics	Methods section 'Participants' and Table 1; https://triad.tnl-mcgill.com/cohort-description/
Recruitment	All subjects in this study were part of the Translational Biomarkers in Aging and Dementia (TRIAD) cohort, a longitudinal imaging and biofluid cohort study of aging and AD. Participants were recruited through advertisements in the community, newspaper advertisements, word of mouth, and referrals from the McGill Centre for Studies in Aging. Evaluations of participants included a review of their medical history and an interview with the participant and their study partner followed by a neurologic examination by a dementia specialist and a neuropsychological examination. The main potential bias is participants' willingness to participate in the study, which may result in cognitive and other aspects of the investigated cohort being different from individuals who are unwilling to participate, or are unaware of this possibility.
Ethics oversight	The McGill University, the Montreal Neurological Institute (MNI) PET working committee, and the Douglas Mental Health University Institute Research Ethics Board (Mental Health and Neuroscience subcommittee of the CIUSSS ODIM REB) provided ethical approval (IUSMD-16-60).<br>All participants gave their written informed consent prior to inclusion in the study. They received a compensation to cover travel expenses and time.

Note that full information on the approval of the study protocol must also be provided in the manuscript.

Field-specific reporting

Please select the one below that is the best fit for your research. If you are not sure, read the appropriate sections before making your selection.

☒ Life sciences ☐ Behavioural & social sciences ☐ Ecological, evolutionary & environmental sciences

For a reference copy of the document with all sections, see nature.com/documents/nr-reporting-summary-flat.pdf

Life sciences study design

All studies must disclose on these points even when the disclosure is negative.

Sample size	All participants that had required data were included in this study, so no specific sample size calculations were performed.
Data exclusions	No data were excluded.
Replication	Overall gradient directions were consistent across subjects representing different stages of the disease; and were in line with literature as published previously for fMRI in healthy controls (Margulies et al 2016 PNAS). We performed additional leave-one-out-cross validation (LOOCV in sklearn, Python). No cohorts other than TRIAD were included in this study.
Randomization	No randomized trials were performed, as allocations into groups were performed by clinical diagnosis (cognitively unimpaired vs impaired [MCI or AD dementia]) and/or amyloid/tau positivity. Amyloid status was determined based on visual rating of the amyloid-PET images, with the final rating based on consensus of two physicians specialized in dementia imaging. Tau status tau status was based on visual rating of the tau-PET images with high retention in early Braak areas (Seibyl et al. 2023 J. Nucl. Med. Off. Publ. Soc. Nucl. Med)

Blinding	Clinical diagnoses were blind to PET results.

Reporting for specific materials, systems and methods

We require information from authors about some types of materials, experimental systems and methods used in many studies. Here, indicate whether each material, system or method listed is relevant to your study. If you are not sure if a list item applies to your research, read the appropriate section before selecting a response.

Materials & experimental systems

n/a	Involved in the study
☒	☐ Antibodies
☒	☐ Eukaryotic cell lines
☒	☐ Palaeontology and archaeology
☒	☐ Animals and other organisms
☒	☐ Clinical data
☒	☐ Dual use research of concern
☒	☐ Plants

Methods

n/a	Involved in the study
☒	☐ ChIP-seq
☒	☐ Flow cytometry
☐	☒ MRI-based neuroimaging

Plants

Seed stocks	*Report on the source of all seed stocks or other plant material used. If applicable, state the seed stock centre and catalogue number. If plant specimens were collected from the field, describe the collection location, date and sampling procedures.*
Novel plant genotypes	*Describe the methods by which all novel plant genotypes were produced. This includes those generated by transgenic approaches, gene editing, chemical/radiation-based mutagenesis and hybridization. For transgenic lines, describe the transformation method, the number of independent lines analyzed and the generation upon which experiments were performed. For gene-edited lines, describe the editor used, the endogenous sequence targeted for editing, the targeting guide RNA sequence (if applicable) and how the editor was applied.*
Authentication	*Describe any authentication procedures for each seed stock used or novel genotype generated. Describe any experiments used to assess the effect of a mutation and, where applicable, how potential secondary effects (e.g. second site T-DNA insertions, mosiacism, off-target gene editing) were examined.*

Magnetic resonance imaging

Experimental design

Design type	resting-state fMRI
Design specifications	n/a
Behavioral performance measures	n/a

Acquisition

Imaging type(s)	Structural T1-weighted MRI, diffusion-weighted MRI, functional (resting-state) MRI, and PET. Dicoms were converted to niftii.
Field strength	3T
Sequence & imaging parameters	T1-weighted MRI images: acquired on a 3T Siemens Magnetom using a volumetric magnetization prepared rapid acquisition gradient echo (MPRAGE) sequence (TR: 2300 ms, TE: 2.96ms).<br><br>PET images: The 18F-MK6240, 18F-NAV4694, and 11C-PBR28 PET scans were acquired at 90-110, 40-70, and 60-90 min following radiotracer injection, respectively, and reconstructed using an ordered-subsets expectation maximization (OSEM) algorithm on a 4D volume with 4 (x300 seconds), 6 (x300 seconds), and 3 (x600 seconds) frames, respectively. They were corrected for dead time, decay, random and scattered coincidences, and attenuation based on a 6-min transmission scan with a rotating 137Cs point source.<br><br>rs-fMRI: collected with single-shot full k-space multiband echo-planar imaging (EPI) and the following parameters: TR = 0.681 s, TE = 32 ms, slice thickness = 2.5mm, number of slices = 54, flip angle = 50 degrees, number of measurements = 870, matrix size: 88 x 88, voxel size = 2.5 mm3 isotropic, and eyes open fixed on a cross.<br><br>diffusion-weighted MRI: collected with EPI sequence and the following parameters: TR = 3500 ms, TE = 71 ms, flip angle = 90 degrees, field of view = 232 x 232 x 162, voxel size = 2 mm3 isotropic, and 13, 48, and 60 isotropically distributed diffusion-sensitizing gradients with b-value = 0, 1000, and 2000 s/mm2, respectively, as well as five b0 images.
Area of acquisition	whole brain

Diffusion MRI ☒ Used ☐ Not used

Parameters: collected with EPI sequence and the following parameters: TR = 3500 ms, TE = 71 ms, flip angle = 90 degrees, field of view = 232 x 232 x 162, voxel size = 2 mm3 isotropic, and 13, 48, and 60 isotropically distributed diffusion-sensitizing gradients with b-value = 0, 1000, and 2000 s/mm2, respectively, as well as five b0 images.

Preprocessing

Preprocessing software	- ICVMapper v1 (https://icvmapp3r.readthedocs.io/en/latest/)<br>- FreeSurfer v7 with PETSurfer<br>- fMRIPrep v.20.2.3<br>- FSL v6.0.5, ANTS v2.3.1, MRtrix3 v3.0.3
Normalization	rigid-body and nonlinear transformations
Normalization template	ADNI template (same space as MNI but template adapted to aging populations with greater atrophy, larger ventricles etc)
Noise and artifact removal	PET: The final smoothing corresponded to 8mm full-width-at-half-maximum of the Gaussian kernel.<br><br>rs-fMRI: Post-processing nuisance regressors were based on the CompCor predefined strategy as outlined in Behzadi et al. and included bandpass filtering (0.01-0.08 Hz), non-steady-state volume, head motion with linear/quadratic terms and derivatives, and six components for the anatomical + temporal CompCors. This was implemented in Python through Nilearn's fmriprep.load_confounds function using the CompCor strategy developed by Wang et al 2023 biorxiv. The rs-fMRI data were smoothed with 4mm full-width-at-half-maximum.<br><br>diffusion-weighted MRI: preprocessed using FSL and MRtrix3, including correction for susceptibility distortions, motion (both between frames and within frames), gibbs ringing, and eddy currents (and removal of the full frame if >20% of the slices within the frame are detected as outlier based on the FSL Eddy report).
Volume censoring	see above.

Statistical modeling & inference

Model type and settings	(partial) correlations, univariate regression, and linear mixed effect models.
Effect(s) tested	correlations between gradients; group-wise differences in gradient scores; associations between gradients and PET/ cognition.

Specify type of analysis: ☐ Whole brain ☐ ROI-based ☒ Both

Anatomical location(s): Cortical nodes were parcellated based on three different brain atlases: (i) an in-house developed high-resolution parcellation based on the multi-modal Glasser atlas re-parcellated into equally-sized sub-regions-of-interest (ROIs) of ~512mm3 totaling 1318 nodes, (ii) the structural-based Desikan-Killiany-Tourville (DKT) atlas implemented in FreeSurfer consisting of 66 nodes, and (iii) the functional-based Schaefer atlas consisting of 100 nodes with addition of the hippocampus based on the Harvard-Oxford atlas.

Statistic type for inference

(See Eklund et al. 2016)

null models using spatial autocorrelation-preserving surrogates

Correction

whole-brain: FWE cluster-wise; gradients: Variogram matching with 1000 permutations; cognitions: FDR.

Models & analysis

n/a	Involved in the study
☐	☒ Functional and/or effective connectivity
☒	☐ Graph analysis
☐	☒ Multivariate modeling or predictive analysis

Functional and/or effective connectivity

undirected weighted connectivity matrix
- fMRI: correlation with Pearson's R
- diffusion MRI: intra-axonal cross-sectional area of the FBC

Multivariate modeling and predictive analysis

Unsupervised dimensionality reduction (https://brainspace.readthedocs.io/en/latest/index.html)
